# Parallelized TCSPC for Dynamic Intravital Fluorescence Lifetime Imaging: Quantifying Neuronal Dysfunction in Neuroinflammation

**DOI:** 10.1371/journal.pone.0060100

**Published:** 2013-04-16

**Authors:** Jan Leo Rinnenthal, Christian Börnchen, Helena Radbruch, Volker Andresen, Agata Mossakowski, Volker Siffrin, Thomas Seelemann, Heinrich Spiecker, Ingrid Moll, Josephine Herz, Anja E. Hauser, Frauke Zipp, Martin J. Behne, Raluca Niesner

**Affiliations:** 1 German Rheumatism Research Center, Berlin, Germany; 2 Max-Delbrück Center for Molecular Medicine, Berlin, Germany; 3 Department of Dermatology and Venerology, University Medical Center Hamburg-Eppendorf, Hamburg, Germany; 4 Charité – University of Medicine, Berlin, Germany; 5 LaVision Biotec GmbH, Bielefeld, Germany; 6 LaVision, Göttingen, Germany; 7 Neurology Department, Johannes Gutenberg University Mainz, Mainz, Germany; Nathan Kline Institute and New York University School of Medicine, United States of America

## Abstract

Two-photon laser-scanning microscopy has revolutionized our view on vital processes by revealing motility and interaction patterns of various cell subsets in hardly accessible organs (e.g. brain) in living animals. However, current technology is still insufficient to elucidate the mechanisms of organ dysfunction as a prerequisite for developing new therapeutic strategies, since it renders only sparse information about the molecular basis of cellular response within tissues in health and disease. In the context of imaging, Förster resonant energy transfer (FRET) is one of the most adequate tools to probe molecular mechanisms of cell function. As a calibration-free technique, fluorescence lifetime imaging (FLIM) is superior for quantifying FRET *in vivo*. Currently, its main limitation is the acquisition speed in the context of deep-tissue 3D and 4D imaging. Here we present a parallelized time-correlated single-photon counting point detector (p-TCSPC) (i) for dynamic single-beam scanning FLIM of large 3D areas on the range of hundreds of milliseconds relevant in the context of immune-induced pathologies as well as (ii) for ultrafast 2D FLIM in the range of tens of milliseconds, a scale relevant for cell physiology. We demonstrate its power in dynamic deep-tissue intravital imaging, as compared to multi-beam scanning time-gated FLIM suitable for fast data acquisition and compared to highly sensitive single-channel TCSPC adequate to detect low fluorescence signals. Using p-TCSPC, 256×256 pixel FLIM maps (300×300 µm^2^) are acquired within 468 ms while 131×131 pixel FLIM maps (75×75 µm^2^) can be acquired every 82 ms in 115 µm depth in the spinal cord of *CerTN L15* mice. The *CerTN L15* mice express a FRET-based Ca-biosensor in certain neuronal subsets. Our new technology allows us to perform time-lapse 3D intravital FLIM (4D FLIM) in the brain stem of *CerTN L15* mice affected by experimental autoimmune encephalomyelitis and, thereby, to truly quantify neuronal dysfunction in neuroinflammation.

## Introduction

The two-photon laser-scanning microscopy (TPLSM) [1] has dramatically changed our perspective on cellular dynamics of both physiologic and pathologic processes especially in the central nervous system and in organs of the immune system [2]–[5], thus, giving the opportunity to develop new diagnostic and therapeutic tools. This is due to the advantages of near-infrared short-pulsed excitation [6]–[7], which enables non-invasive tracking of cellular motility and communication deep within living organisms, i.e. in intravital imaging. However, the molecular basis of cellular function, the key to organ function, remains difficult to access in dynamic highly-resolved intravital studies.

The most common phenomenon used to monitor cellular function on a molecular basis in optical imaging is Förster Resonant Energy Transfer (FRET), which has been extensively employed *in vitro* to probe protein-protein interactions and examine cellular phenomena [8]–[12]. The development of mouse models encoding FRET biosensors based on fluorescent proteins [13] has opened new opportunities for the long-term monitoring of cell and tissue function in living organisms [14]–[17].

Numerous technological advances are required to fully take advantage of the FRET potential for intravital quantification of cell function. In addition to the improvement of microscope optical performance and the development of small animal surgical techniques with the general aim of non-invasive imaging, calibration-free signal quantification and fast 3D-image acquisition are needed, especially to allow observation of pathologic changes of tissue in response to interactions with highly motile cells (e.g. those moving at a rate of 5–7 µm/min) [2]–[4]. As far as physiological modifications are concerned, even higher acquisition rates are required, for instance, calcium oscillations take place on the time scale of a few milliseconds.

Established quantitative FRET techniques include [8]–[9]:

- different ratiometric techniques [18],
- photobleaching-based techniques [10], [19]
- fluorescence lifetime imaging (FLIM) [11], [20]–[21] and
- time-resolved fluorescence anisotropy imaging (trFAIM) [11].

Photobleaching-based techniques are very reliable, but rather slow and potentially invasive. Although calibration-free, trFAIM leads to slight read-out modifications at low signal strength, which are incompatible with the requirements of deep-tissue imaging. While ratiometric FRET techniques have already been applied intravitally [3], [14], [18], in order to fulfill the requirements for dynamic, calibration-free intravital FRET quantification, fast and accurate FLIM with high spatial resolution, deep within the tissue is needed. Although faster than FLIM, ratiometric techniques are prone to artifacts due to light scattering caused by strong refractive index mismatches in tissue. Scattering is dependent on the wavelength (emission/excitation) [22], thus, inducing a different degree of deterioration regarding resolution and signal-to-noise ratio (SNR) for different emitted wavelengths at increasing imaging depth. Concretely, the shorter wavelength emission of the donor and the longer wavelength emission of the acceptor of the FRET-pair are characterized by different depth-dependent signals and SNR which induce an artificial depth-dependent gradient of the FRET-ratio.

Because of the multi-exponential nature of the fluorescence signal typically acquired in tissue, the use of time-domain FLIM techniques is recommended [23]. Multifocal scanning combined with field-detection [24], especially of gated optical intensifiers (GOI), ensures fast, video-rate FLIM [25]. Deficits of this approach include intrinsically poor photon economy (∼10% signal exploitation), relatively poor resolution and limited imaging depth in tissue. The alternative use of streak cameras, which allow direct fluorescence decay acquisition, and thus presumably better photon economy, is limited by the slow speed of photon counting electronics, typically 1 MHz [26].

The most accurate techniques which allow for diffraction-limited FLIM [12] are based on time-correlated single-photon counting (TCSPC). Intravital FRET-FLIM microscopy and microendoscopy [12], [17] have already been applied in cancer research. However, the principal deficit of standard TCSPC remains the poor photon economy, due to slow photon counting electronics. Count rates are typically 8 MHz for time-to-amplitude-converters (TAC) [27]–[28] or 11 MHz for time-to-digital-converters (TDC) [29], whereas the repetition rate of TPLSM lasers is typically 80 MHz. This leads to slow image acquisition, up to 90% signal loss and potentially to photobleaching and tissue photodamage. In contrast, multifocal TPLSM combined with TCSPC based on 16 channel multi-anode photomultiplier tubes (PMT) is able to increase the acquisition speed in 3D-FLIM, and has been applied to probe NAD(P)H metabolism in a cancer cell line [27]. However, this approach is still limited by slow electronics, with an average count rate of 3.2 MHz, and by considerable photobleaching (18% between successive 3D images) [27]. Scalable multichannel TCSPC systems based on TDC, which increase the average count rate, have been employed in fluorescence correlation spectroscopy (FCS) rather than FLIM [29]. Using a parallelized TCSPC technique with different detectors and electronics compared to our technology, a FLIM acquisition speed of 100 ms/ 100×100 pixel (10 µs/pixel) has been reported at the surface of brain slices [30]. Multi-parametric, fast wide-field FLIM has been used to quantify protein-protein interactions in living cells [31]. As far as the sensitivity is concerned, hybrid detectors used in new FLIM systems are a very promising solution for fast highly sensitive FLIM.

Still, fast dynamic 3D-FLIM (4D-FLIM) deep within hardly accessible organs of living animals as required by typical biological questions has not been yet demonstrated.

As far as the analysis of the largely unknown multi-exponential fluorescence decays in tissue is concerned, reliable and fast algorithms have been adapted for FLIM, e.g. Phasor-approach [32], stretched-exponential [33], Laguerre expansion [34] or genetic algorithms [20] in addition to the standard Levenberg-Marquardt iterative approach.

Here we present a highly efficient 16-channel parallelized TCSPC (p-TCSPC) technique with an average count rate of 80 MHz, which allows fast fluorescence lifetime map acquisition limited only by the frequency of the galvoscanner, i.e. 512×512 pixel lifetime images are acquired within 427 ms (1.63 µs/pixel). Since the main requirement in live tissue and intravital FLIM with relevance for cell biology or the investigation of (immune-induced) pathologies is fast acquisition in the range of tens or hundreds of milliseconds, we compare the performance of our new device to multifocal GOI, i.e. the standard technique for ultra-fast video-rate FLIM, and to highly sensitive single-channel TCSPC using hybrid detectors both on standardized dye solutions and in live samples (hippocampus slices and brain stem of live mice). Thereby, special focus is dedicated to the relevance of parallelization of detection and counting electronics in order to increse acquisition speed in dynamic intravital FLIM.

By employing p-TCSPC, we are for the first time able to dynamically quantify neuronal function via calcium levels in the chronically inflamed brain stem of *CerTN L15* mice [14]. We chose experimental autoimmune encephalomyelitis, a murine model of Multiple Sclerosis, to induce chronic inflammation to the central nervous system (CNS). We thus demonstrate the practical value of this technique as a tool to truly quantify the cellular mechanisms of early neural injury occurring in CNS inflammation.

## Materials and Methods

### Two-photon laser-scanning microscopy (TPLSM)

FLIM experiments were performed using a specialized two-photon laser-scanning microscope (Fig. S1a) based on a commercial scan head (TriMScope, LaVision BioTec, Bielefeld, Germany) [35]–[36]. The novel 16-channel TCSPC detector (FLIM-X_16_, LaVision BioTec, Bielefeld, Germany) and the hybrid-detector single-channel TCSPC (HPM-100-40 combined with a 10 MHz SPC-150, Becker&Hickl, Berlin, Germany) were used in combination with single-beam laser-scanning whereas the time-gated FLIM field-detector (PicoStar, LaVision, Göttingen, Germany) was used with multi-beam (up to 64 beams) scanning. For detailed setup descriptions see *Material S1*.

### Mice

The *CerTN L15* mouse expresses a FRET-based calcium biosensor consisting of Cerulean (donor) and Citrine (acceptor) bound to Troponin C, a calcium-sensitive protein in certain subsets of neurons [14]. Similar, the Thy1 EGFP mouse expresses EGFP (enhanced green fluorescent protein) in certain subsets of neurons. The ActRFP mouse expresses tdRFP (tandem red fluorescent protein) ubiquitously. Further description and handling of the *CerTN L15* mouse, *Thy1 EGFP* mouse (JAX Laboratory) and *acRFP* mouse can be found in *Material S1*.

### Brain slice preparation

Brains of *CerTN L15* or *Thy1 EGFP* 6–8 week old mice were removed and immediately put into 4°C aerated (carbogen, 95% O_2_ and 5% CO_2_) artificial cerebrospinal fluid (ACSF) containing 124 mM NaCl, 1.25 mM NaH_2_PO_4_, 26 mM NaHCO_3_, 3 mM KCl, 1.6 mM CaCl_2_, 1.8 mM MgSO_4_ and 10 mM glucose, adjusted to pH 7.35. 300 μm-thick brain slices were cut with a Vibratome (VT 1200 S, Leica). Hippocampus slices were isolated, allowed to recover for at least 45 min at room temperature before they were transferred to a heated custom-made slice chamber (37°C), in which slices were continuously perfused with previously warmed carbogen-aerated ACSF.

### Preparation of the brain stem and spinal cord window for intravital imaging

The preparation of the imaging field was similar to our previous description [3]. For a detailed description see *Material S1*. Animal experiments were approved by the appropriate state committees for animal welfare (LAGeSo, Landesamt für Gesundheit und Soziales) and were performed in accordance with current guidelines and regulations.

### Data analysis

Evaluation of the multi-exponential fluorescence signal was performed by the self-developed software RINIFLIM programmed in Matlab (MathWorks, USA). For evaluation, Levenberg-Marquardt algorithms or global analysis were used (*Material S1*). The here presented technology can be used in combination with the phasor approach as demonstrated [37].

## Results

### FLIM benchmarking: accuracy vs. acquisition speed

We compared the accuracy and acquisition speed of the p-TCSPC device to that of the GOI and of the hybrid-detector based single-channel TCSPC (single-channel TCSPC). The accuracy of fluorescence decays in an image is given by the maximum and the full-width-at-half-maximum (FWHM) of the Gaussian approximated distributions of the fluorescence lifetimes and of the prefactors.

The mono-exponential fluorescence decay image (455×455 pixel, 150×150 µm^2^) of a 100 µM Rhodamine 6G (Rh6G) solution in water is acquired by the GOI within 88 ms, limited only by the read-out time of the camera (PixelFly, 2× binning, 43.5 ms). The acquisition time of a mono-exponential fluorescence decay image (512×512 pixel, 150×150 µm^2^) of the same solution performed with the p-TCSPC setup amounts to 427 ms, which is considerably shorter than that of the high-performance single-channel TCSPC, i.e. 3 s acquisition time to avoid pile-up. If the number of detected photons (for 10 MHz, approx. 10^6^ photons/s) exceeds the maximum number of events that can be evaluated by the counting electronics, an artificial shortening of the fluorescence lifetime is induced, termed “pile up”. The fluorescence lifetime values amount to 4184 ps (71 ps FWHM) for GOI, 4121 ps (123 ps FWHM) for p-TCSPC and 4049 ps (98 ps FWHM) for the single-channel TCSPC, which corresponds with published results, i.e. 4080 ps [38].

Since applications in the life sciences especially in TPLSM are related to rather low fluorescence signals, we further compared the performance of the FLIM devices on a more diluted 10 µM solution of Rh6G (for mono-exponential decay) (Table 1, *Fig. S2*) and on different mixtures of 10 µM stock solutions of Rhodamine B (RhB) and Rh6G (for bi-exponential decay) (Table 1, *Fig. S1c and S1d*). The results listed in *Table* *1* correspond to the expected lifetime values for Rh6G and RhB, i.e. 1700 ps [38]–[39]. Also the expected relative concentrations *a_1_* and *a_2_* of RhB (short lifetime τ_1_) and Rh6G (long lifetime τ_2_), respectively, are accurately restored by the bi-exponential evaluation (*Table* *1*). The experimental parameters, i.e. acquisition times, peak photon fluxes of the excitation laser at the sample and image dimensions are included to Table 1.

**Table 1:** Mono- and bi-exponential FLIM benchmarking experiments on standardized dyes.

dye conc.	vol. fraction	GOI					pTCSPC					single channel TCSPC				
		100×100 pixel, 33×33 µm^2^					100×100 pixel, 29×29 µm^2^					100×100 pixel, 29×29 µm^2^				
		ph./pixel	τ_1_ /ps	τ_2_ /ps	a_1_/a_1_+a_2_/%	?^2^_R_	ph./pixel	τ_1_ /ps	τ_2_ /ps	a_1_/a_1_+a_2_/%	?^2^_R_	ph./pixel	τ_1_ /ps	τ_2_ /ps	a_1_/a_1_+a_2_/%	?^2^_R_
Rh6G	-	920	-	4003	-	1.26	860	-	4028	-	1.02	890	-	4049	-	1.09
				(83)					(79)					(136)		
RhB	-	850	1691	-	-	1.35	730	1724	-	-	0.94	966	1740	-	-	1.15
			(77)					(53)					(48)			
acq time		4.95 s					8.6 s					15 s				
ph. flux		4.57·10^29^ photons/s·cm^2^					4.14·10^29^ photons/s·cm^2^					8.28·10^28^ photons/s·cm^2^				
count rate		9.8·10^5^ photons/s					8,8·10^5^ photons/s					4.6·10^5^ photons/s				
RhB:Rh6G	4:1	1080	1750	4150	83.3	1.36	1100	1712	4182	81.2	0.99	-	-	-	-	-
			(268)	(281)	(3.5)			(98)	(199)	(2.8)						
RhB:Rh6G	1:1	1360	1811	4158	53.3	1.28	1260	1773	4097	52.3	1.02	1250	1738	3972	53.7	1.22
			(283)	(226)	(5.2)			(123)	(160)	(3.1)			(192)	(224)	(4.8)	
aqc time		9.9 s					10.6 s					15 s				
ph. flux		4.57·10^29^ photons/s·cm^2^					4.14·10^29^ photons/s·cm^2^					8.28·10^28^ photons/s·cm^2^				
count rate		1.2·10^6^ photons/s					1.05·10^6^ photons/s					5.7·10^5^ photons/s				

The values in brackets represent FWHM (full width at half maximum) of the corresponding distribution of the parameters τ_1_, τ_2_, a_1_/(a_1_+a_2_), respectively, in the (mono- or bi-) exponential approximations. The experimental parameters, i.e. frame dimension, photon flux, acquisition time and count rate, are given for each FLIM detector. λ_exc_  = 800 nm, λ_emission_  = 593±20 nm.

### Spatial resolution: diffraction-limited fluorescence lifetime images

The spatial resolution is given by the effective point spread function (ePSF), which was measured as described elsewhere (*Material S1*, [35]–[36], [40]). Whereas the axial resolution is the same for both single-beam and multi-beam setup, the lateral resolution of GOI is inferior by 2.2 fold when compared to p-TCSPC (*Fig. S3*) under similar excitation conditions. The resolution of both single-channel TCSPC and p-TCSPC setups (350±20 nm lateral, 1310±160 nm axial, s.d.) at λ_exc_  = 800 nm is consistent with the result of the paraxial approximation: 370 nm lateral, 1329 nm axial [40]. Scattering in deep-tissue leads to deterioration of spatial resolution with increasing imaging depth [35]–[36], [41]. Due to the fact that scattering scales with λ^-2^ with λ the (emission or excitation) wavelength [22] and that the – shorter wavelength – emission influences only the resolution on field detectors, this effect is more pronounced on the GOI than the TCSPC detectors (single-channel TCSPC or p-TCSPC) (*Material S1*, *Fig. S3*).

### Depth-dependent SNR (ddSNR) and maximal imaging depth in FLIM

In steady-state TPLSM, the maximal imaging depth is reached when the depth-dependent SNR (ddSNR) becomes one. In time-resolved experiments, the accuracy of the FLIM results deteriorates due to scattering within tissue and limits the real imaging depth, expressed as the increase of lifetime distribution width. Comparing the performance of the field-detection FLIM setup (GOI) with the point-detection devices (p-TCSPC or single-channel TCSPC) on porcine skin biopsies stained with fluorescein-isothio-cyanate (FITC) as an example of optically dense, scattering tissue, and in brain slices of *Thy1 EGFP* mice as an example of homogeneous tissue reveals the maximal imaging depth given by ddSNR to be larger than the FLIM depth. The reasons for this discrepancy are fundamentally different for the GOI setup as compared to the TCSPC setups, as discussed later. We heuristically set the FLIM accuracy limit to a lifetime distribution width of 1000 ps, at which the mean fluorescence lifetime deviates considerably from the expected value of FITC (3600 ps) and EGFP (2600 ps) [42]–[43].

**Figure 1:**
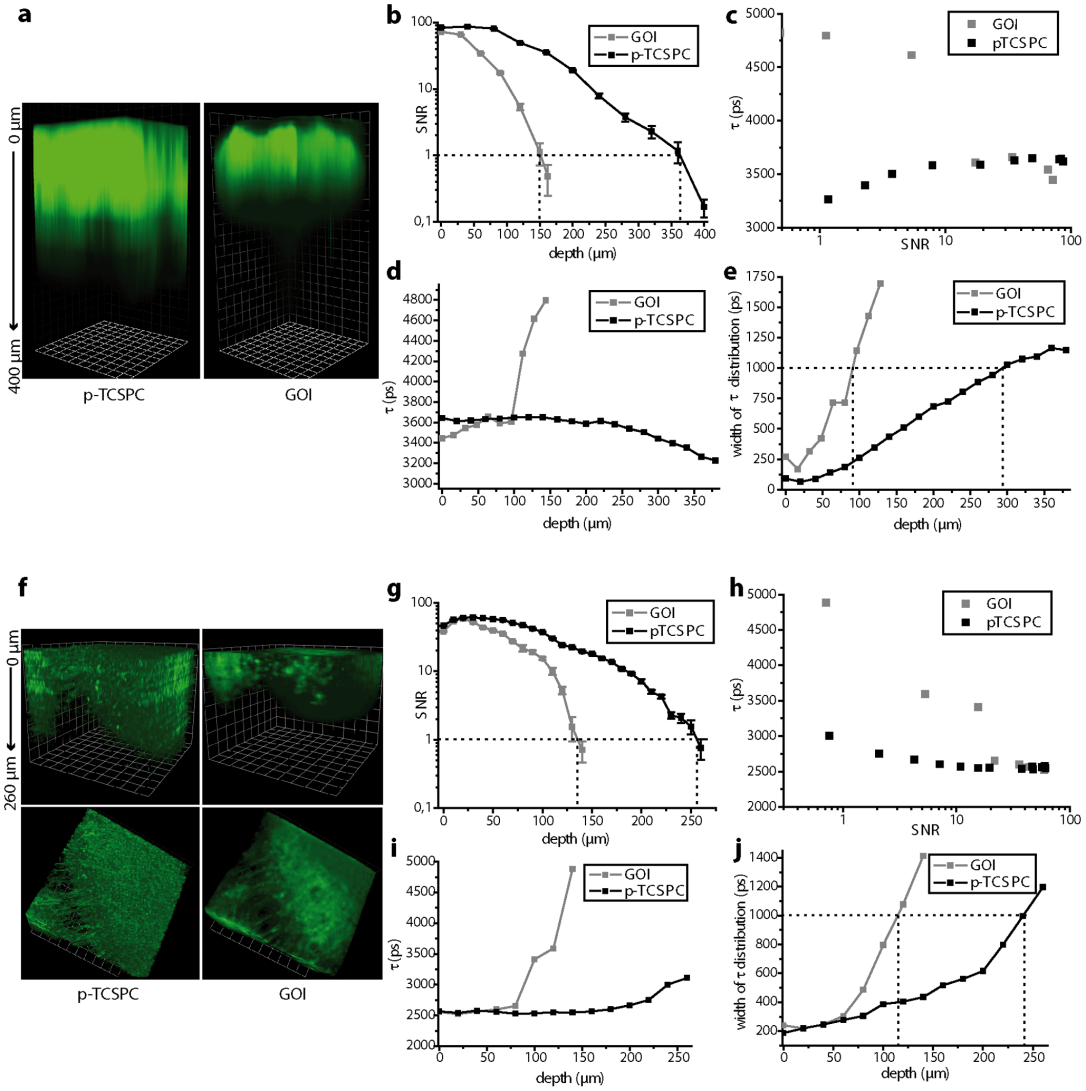
Depth-dependent SNR and maximum imaging depth in FLIM. Three dimensional fluorescence images acquired by means of the GOI and p-TCSPC setup, respectively, at the same region **(a)** of a fluorescein-isothio-cyanate (FITC) stained skin biopsy (200×200×400 µm^3^) and **(f)** of a hippocampal slice of a *Thy1 EGFP* mouse, in which neuronal subsets express EGFP, (300×300×260 µm^3^). Corresponding depth dependent signal-to-noise ratio (ddSNR) curves are shown in **(b)** for the skin biopsy and in **(g)** for the hippocampal slice**.** Dependence of the mean fluorescence lifetime on ddSNR is depicted in **(c)** for the dermal samples and in **(h)** for the hippocampal slices. Depth dependence of the mean fluorescence lifetime of FITC in the skin biopsy is depicted in **(d)** and of EGFP in the hippocampal slice is shown in **(i)**. The corresponding widths (Gaussian full-width-at-half-maximum) of the lifetime distributions are shown in **(e)** and **(j)**, respectively. Setup parameters are listed in *Material S1*.

Under these conditions the ddSNR imaging depth at the same region of a FITC-stained skin biopsy at similar laser power per beam and similar exposure time (*Material S1*) was 150 µm with the GOI setup and 365 µm with the p-TCSPC setup, an increase of 2.43-fold (Fig. 1a, b). The FLIM depth at the same area amounted to 90 µm with the GOI setup as compared to 290 µm with the p-TCSPC setup (Fig. 1d, e). Interestingly, considerable deviation from the expected mean fluorescence lifetime of FITC occurs already at a ddSNR larger than 10 for the GOI setup as compared to a ddSNR value of 4 for the p-TCSPC setup (Fig. 1c). We explain this observation by the allocation of emitted photons in false pixels on the GOI due to scattering effects, which on its turn leads to an apparent time-spread of the photon arrival. In hippocampal slices of *Thy1 EGFP* mice the ddSNR imaging depth under similar excitation conditions (*Material S1*) and at the very same area amounted to 133 µm with the GOI setup and 258 µm with the p-TCSPC setup, an increase of 1.94-fold (Fig. 1e, f) whereas the FLIM depths were 115 µm and 240 µm (Fig. 1g, h), respectively. The dependence of the mean fluorescence lifetime on ddSNR depicted in Fig. 1 h emphasizes that also in brain slices strong deviations from the expected fluorescence lifetime occur for the GOI setup already at ddSNR of 17 whereas for the p-TCSPC setup the critical ddSNR is much lower (between 3 and 4).

The imaging depths, i.e. ddSNR depth and FLIM depth, attained by means of single-channel TCSPC were similar to those reached if using the p-TCSPC setup. Thereby, the excitation power/excitation photon flux was reduced up to 5 times in the single-channel TCSPC setup as compared to the p-TCSPC setup to avoid pile-up (max. 10^6^ photons/s).

In favor of avoiding artifacts caused by a more complicated results interpretation we omitted the widely used exponential increase of mean excitation power to increase imaging depth in tissue [6].

### Calcium imaging by 4D-FRET-FLIM in *CerTN L15* mice: performance of p-TCSPC vs. GOI in hippocampus slices

Quantitatively monitoring cellular function by FRET directly in living tissue is pivotal to fully understand basic physiologic and pathologic mechanisms. Therefore, time-lapse 2D and 3D FRET-FLIM, ensuring low photobleaching and photodamage, high acquisition speed and sub-cellular resolution deep within tissue, is required.

We compared the single-beam p-TCSCP to the multifocal GOI setups by probing neuronal function in acute hippocampal slices of adult *CerTN L15* mice during membrane depolarization induced by increased K^+^ ion concentration (maximal FRET efficiency 65%). Brain slices prepared in this manner have been shown to retain a layer of living tissue in the middle of the slice, which is comparable to the native central nervous system (CNS) environment [44]. Imaging was performed in this living layer of acute slices, beyond the glial scar, starting at a depth of 30 µm.

**Figure 2:**
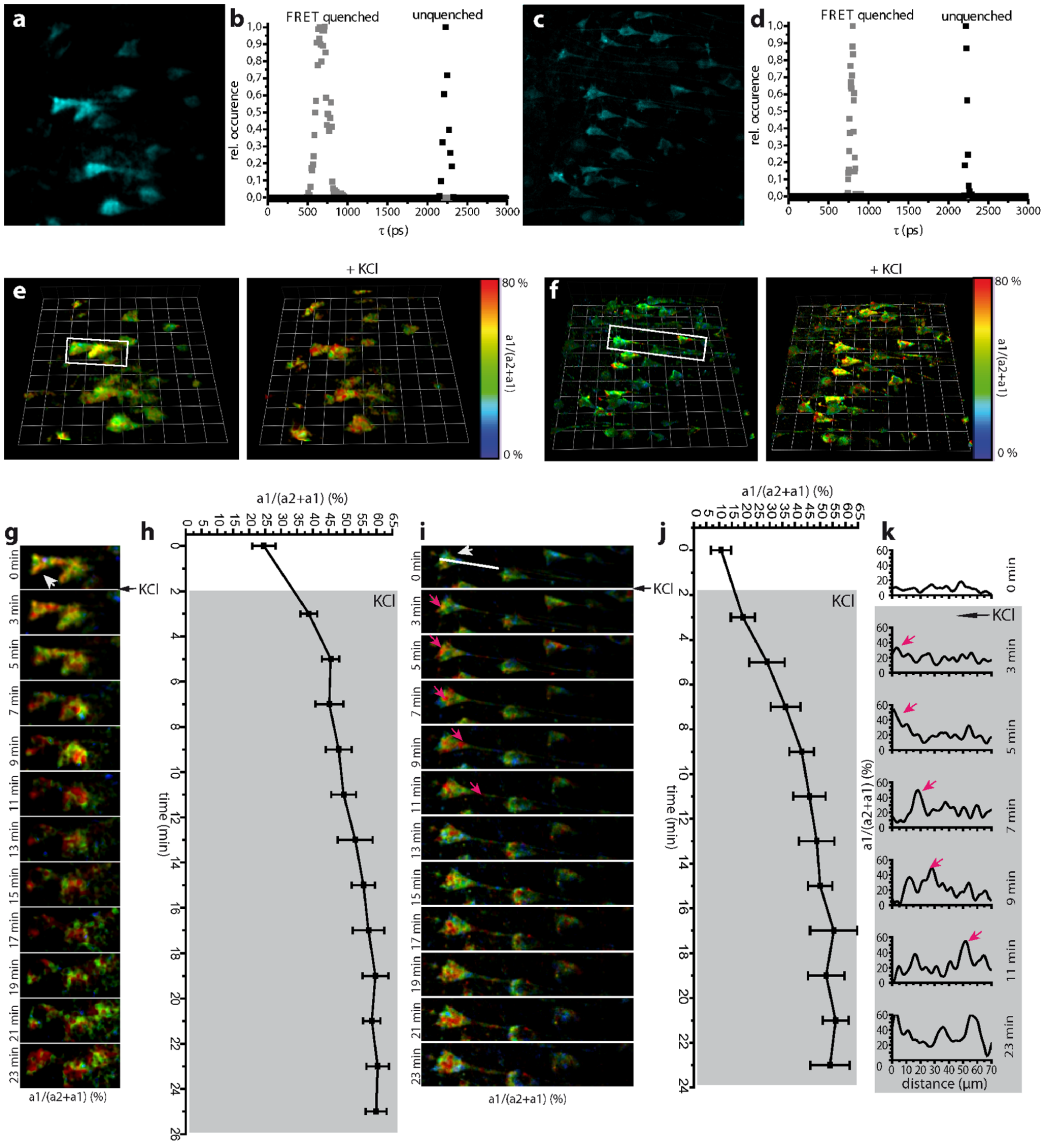
Dynamic FRET-FLIM in living hippocampal slices of *CerTN L15* mice. Projections of three dimensional (3D) fluorescence data sets **(a)** (221×221×12 µm^3^, 492×492×5 voxel) acquired by the GOI setup and **(c)** (200×200×12 µm^3^, 512×512×5 voxel) acquired by the p-TCSPC setup in a living hippocampal slice of a *CerTN L15* mouse. The fluorescence lifetime distributions of the FRET-quenched and unquenched Cerulean corresponding to **(a)** and **(c)** are depicted in the graphs **(d)** and **(b)**. **(e)** and **(f)** show corresponding 3D a_1_·100/(a_2_+a_1_) images (FRET-ratio images) before and 7 minutes after perfusion with a 100 mM KCl solution as acquired with the GOI and p-TCSPC setup, respectively. The depolarization of the neurons in the presence of highly concentrated K^+^ ions leads to an increase of neuronal calcium level, as shown by increased FRET signal. **(g)** Time-lapse images showing neurons in the demarcated area in **(e)** depicting the increase of calcium in neuronal somata. The graph **(h)** shows the increase of calcium level in the soma indicated in **(g)** by the arrowhead. 3D unit  = 28 µm, scale bar  = 20 µm. **(i)** Time-lapse showing neurons in the demarcated area in **(f)** depicting the increase of calcium in neuronal somata and processes. The magenta arrows in (**i**) indicate the incomplete depolarization immediately after application, i.e. high calcium levels are still led from the dendrites through the soma to the axon before the neuron is completely flooded by calcium. The graphs in **(k)** show the neuronal calcium level at the indicated time points along the white line in **(i),** reaching from the soma to the axon. The magenta arrows in (**k**) correspond to the Calcium increase also indicated by magenta arrows in the image series (**i**). The same process of neuronal depolarization as in **(h)** for the GOI setup can be observed in **(j)** for the p-TCSPC setup. 3D unit  = 30 µm, scale bar  = 20 µm. Imaging was performed starting from 15–30 µm depth in tissue, which is beyond the glial scar.

The fluorescence lifetimes of unquenched and quenched Cerulean amount to 2225 ps (22 ps FWHM) and 693 ps (45 ps FWHM) with the p-TCSPC detector (Fig. 2b) and to 2234 ps (61 ps FWHM) and 677 ps (139 ps FWHM) with the GOI detector (Fig. 2d). This corresponds to previously determined values within the error margins [45]. The fluorescence lifetimes of both unquenched and FRET-quenched Cerulean in *TN L15* measured here in brain tissue are somewhat shorter than those measured under extracellular conditions by us (τ_unquenched_  = 2491 ps (158 ps FWHM), τ_FRET-quenched_  = 808 ps (49 ps FWHM)) and by others [46]. We attribute this to differences in refractive index *n*, i.e. the fluorescence lifetime τ scales with 1/*n* ^2^ [47]. The lifetime values did not change over the whole acquisition period (40 time points), therefore excluding the possibility of eventual photo-isomerization of Cerulean, as has been reported for different conditions [48]. No significant change in mean fluorescence lifetime was observed down to 100 µm imaging depth in brain tissue.

**Figure 3:**
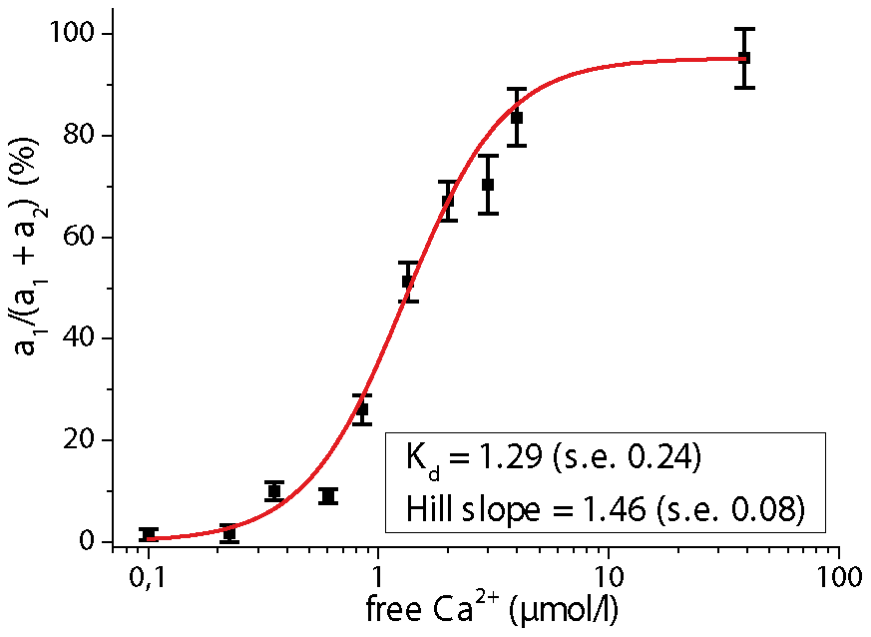
FLIM-based Calcium calibration of TN L15. The FRET signal of TN L15 (Troponin C bound to the FRET pair Cerulean and Citrine) was measured by FLIM in buffered solutions of different free Calcium concentrations in the range 0 µM to 39 µM (Ca Calibration Buffer Kit, Invitrogen, Germany). The fluorescence decays were biexponentially approximated. In all cases, the fluorescence lifetime of the FRET-quenched Cerulean amounts to 808 ps and that of the unquenched Cerulean to 2491 ps. These values well agree according to the Strickler-Berg dependence on refractive index to the values measured in brain slices and in the brain stem of live mice. The ratio a1/(a1+a2) of FRET-quenched to unquenched Cerulean represents the FRET signal and is depicted on the ordinate. The inset shows the values for K_d_ and the Hill slope.

A 3D image restoring the ratio a_1_·100/(a_2_ +a_1_) gives the relative concentration of FRET quenched to total Cerulean concentration. The FRET signal shows a sigmoid dependence on calcium concentration (apparent K_d_  = 1.29±0.24 µM, Hill slope  = 1.46±0.08, (Fig. 3) These values confirm previous calibration of the TN L15 construct (K_d_  = 1.2, Hill slope  = 1.47) performed by ratiometric FRET [49].

The time-step for a similar time-resolved 3D data set of Cerulean, i.e. 492×492×5 voxel with the GOI setup (Fig. 2a) and 512×512×5 voxel with the p-TCSPC setup (Fig. 2c), respectively, was set to 120 s. The time-step does not correspond to the true acquisition time required to collect enough photons for the bilinear FRET-FLIM evaluation. We limited the acquisition rate to minimize photobleaching and photodamage if using the GOI setup and, thus, to enable time-lapse FLIM with the multifocal GOI setup. A reduction of the excitation power in the GOI setup led to fluorescence signals that are too low to be further evaluated. The peak photon flux was similar for both detectors (*Material S1*) and was adjusted in a way that at least 1000 photons/pixel are acquired. The true acquisition time for a 492×492×5 voxel image was 41.9 s with the GOI setup and for a 512×512×5 voxel image was 25 s with the p-TCSPC setup (19 µs/voxel)

The FRET signal in neurons within the middle layer typically amounts to 9±3% (N = 11 cells, s.d.), i.e. 240±27 nM calcium, with the p-TCSPC and to 17±6% (N = 7 cells, s.d.), i.e. 417±53 nM calcium, with the GOI. The damaged superficial neurons in the glial scar can locally reach values of up to 52%, i.e. 1.39 µM calcium, as depicted in measurements performed with the p-TCSPC (Fig. 2f).

We monitored the calcium distribution in neurons before and during perfusion with 100 mM KCl solution (Fig. 2e, f, *movies S1* and *S2*). Using both the GOI and the p-TCSPC setup, we observed an increase of mean calcium concentration in neurons due to membrane depolarization (Fig. 2g, i). However, the sub-cellular distribution of pathologically caused calcium waves over time could only be visualized using the p-TCSPC setup (Fig. 2i). Here it became evident that during membrane depolarization via increased K^+^ a slow propagation of a high amplitude calcium wave (single peaks between 35% and 62% FRET ratio) from the dendrites through the soma to the axon, similar to the fast calcium oscillation under physiological conditions, overlapped a slight overall increase of the calcium level (9±3% before as compared to 18±4% immediately after KCl addition). After approx. 20 minutes the neurons were completely flooded by calcium (65% FRET-quenched Cerulean, i.e. 1.97 µM Ca^2+^). This calcium level increase in neurons correlates with changes in neuron morphology: over time, both the somata and the cell nuclei showed signs of swelling before the cell membrane disintegrated.

As far as intravital FLIM is concerned, the multifocal GOI setup was not able to repeatedly recover enough fluorescence signal due to the need for an extremely high excitation power, which led to photobleaching after very few illumination steps.

### Fast and ultra-fast intravital calcium imaging by 3D-FRET-FLIM in *CerTN L15* mice: performance of p-TCSPC vs. single-channel TCSPC in the spinal cord

**Figure 4:**
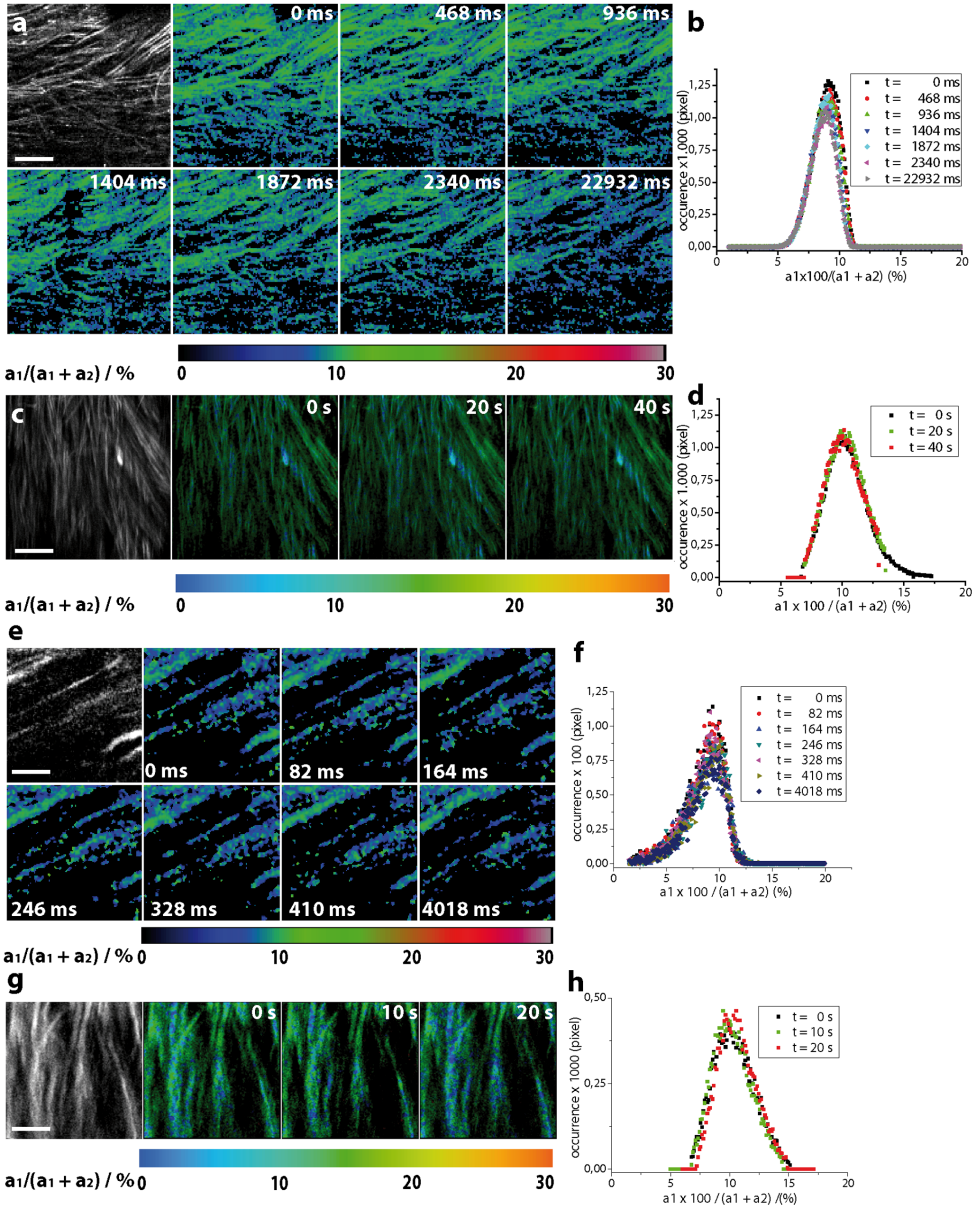
Dynamic intravital FRET-FLIM in the spinal cord of healthy *CerTN L15* mice. **(a)** Intensity of Cerulean and FRET-ratio (a_1_·100/(a_1_ + a_2_) ratio) maps of axons in the spinal cord of a CerTN L15 mouse. 300×300 µm^2^ (256×256 pixel) FRET-FLIM images are acquired every 468 ms using the p-TCSPC device. **(b)** The accuracy of the FRET-ratio in these data is quantified by the width of its distribution over an image (e.g. 9.04±1.11% corresponding to 242±76 nM calcium, t = 0 ms). This value corresponds to the expected calcium concentration in healthy neurons (44). The distributions of the FRET-ratio do not change over time (after 50 illumination steps). **(c)** Intensity of Cerulean and FRET-ratio maps (300×300 µm^2^, 256×256 pixel) of axons in the spinal cord of a CerTN L15 mouse must be acquired every 20 s using the high-performance single-channel TCSPC to avoid pile up effects (maximally 10^6^ photons/s) which artificially reduce the fluorescence lifetime of Cerulean and increase the FRET-ratio, i.e. the apparent calcium concentration. Thus, a much lower excitation power (1.8 mW instead of 18 mW as used in the p-TCSPC setup at 850 nm excitation wavelength) was applied. The full potential of the hybrid detector could not be exploited due to the limited average counting rate of the electronics. **(d)** The distributions of the FRET-ratio corresponding to the images in (c) are similar to the distributions measured using the p-TCSPC setup. Thus, the accuracy of the FRET-ratio measured by p-TCSPC (a) and single-channel TCSPC (c) is also similar. **(e)** 75×75 µm^2^ (131×131 pixel) FRET-ratio maps in the spinal cord of the same mouse line could be acquired every 82 ms using the p-TCSPC device. **(f)** The accuracy of the images in (e) is restored by the distribution of the FRET-ratio (9.09±1.58% corresponding to 244±10.6 nM calcium, t = 0 ms), which remains stable over time (50 illumination steps). **(g)** FRET-ratio maps of the same dimensions (75×75 µm^2^, 131×131 pixel) must be acquired every 10 s in order to simultaneously avoid pile-up effects and to achieve the same accuracy as by p-TCSPC-FLIM. **(h)** Distributions of FRET-ration in the images in (g).

We compared the novel p-TCSCP (80 MHz average counting rate) with a hybrid-detector based single-channel TCSPC setup (10 MHz average counting rate) by probing neuronal function in the spinal cord of healthy *CerTN L15* mice. Imaging was performed in approx. 115–130 µm depth (Fig. 4).

We focused on (2D+time)-FRET-FLIM over large fields of view (300×300 µm^2^, 256×256 pixel) with relevance in the investigation of pathologies. Using the p-TCSPC setup we could acquire a FRET-ratio image by FLIM every 468 ms (2000 photons/pixel, 18 mW (peak photon flux *Ø*  = 4.36·10^30^ photons/s·cm^2^, λ_exc_  = 850 nm, count rate 6.38·10^7^ photons/s). The accuracy of the images is restored by the parameters of the FRET-ratio distribution (t = 0 ms, 9.04±1.11% corresponding to 242±8.03 nM calcium). Even after 50 illumination steps the accuracy is retained (t = 22932 ms, 8.57±1.00% corresponding to 226±7.6 nM). In order to avoid pile-up effects, which induce an artificial shortening of the fluorescence lifetime and, thus, an increase of the FRET-ratio, i.e. of the apparent calcium concentration we limited the excitation power when using the single-channel TCSPC setup to 1.8 mW (peak photon flux *Ø*  = 4.36·10^29^ photons/s·cm^2^, λ_exc_  = 850 nm, count rate 1.6·10^6^ photons/s). Under these conditions, 20 s were necessary to acquire a 300×300 µm^2^ (256×256 pixel) FRET-ratio map (2460 photons/pixel). The accuracy of the FRET-ratio is similar to that achieved by the p-TCSPC setup (t = 0 s, 10.04±1.58% corresponding to 271±10.6 nM calcium).

Ultra-fast (2D+time) FRET-FLIM on the time-scale of milliseconds (75×75 µm^2^, 131×131 pixel) with relevance for cell physiology and cell biology emphasizes the superiority of electronics and detector parallelization (p-TCSPC) as compared to high-performance single-channel TCSPC (Fig. 4. e-h). Using the p-TCSPC setup we could acquire a FRET-ratio image by FLIM every 82 ms (1700 photons/pixel, 12 mW (peak photon flux *Ø*  =  2.9·10^30^ photons/s·cm^2^, λ_exc_  = 850 nm, count rate 4.2·10^7^ photons/s). The accuracy of the images is given at t = 0 ms by the FRET-ratio distribution 9.09±1.58% corresponding to 244±10.6 nM calcium. Even after 50 illumination steps the accuracy is retained (t = 4018 ms, 9.04±1.59% corresponding to 242±10.6 nM). Using the single-channel TCSPC at 0.9 mW excitation power and 850 nm excitation wavelength, 10 s were needed to acquire a FRET-ratio map of 75×75 µm^2^ (131×131 pixel). The count rate amounted to 1.05·10^6^ photons/s. The accuracy in this case was also similar to that of the p-TCSPC (2100 photons/pixel, t = 0 s, 10.25±1.81% corresponding to 276±11.7 nM calcium). In this context, it has to be mentioned that the use of the FRET-based constructs for such questions is rather inadequate, since the response time of the constructs to the stimulus, in this case calcium, is lower (520 ms) than the frame time of FLIM and the time resolution typically required by questions in cell biology [15], [49]. Furthermore, the dynamic range (K_d_ and Hill slope) of the construct must be considered (Fig. 3) when choosing the genetically encoded calcium construct to answer a specific question.

The fluorescence lifetimes of unquenched and FRET-quenched Cerulean restored by biexponential approximation of the FLIM data acquired with single-channel TCSPC (*Fig. S4*) typically amounted to 2269 ps (FWHM 214 ps) and 754 ps (FWHM 158 ps), respectively (χ^2^  = 1.00). Using the p-TCSPC, the fluorescence lifetimes of unquenched and quenched Cerulean amounted to 2316 ps (223 ps FWHM) and 778 ps (115 ps FWHM), respectively (χ^2^  = 1.09).

### Intravital quantification of neuronal calcium in CNS inflammation by p-TCSPC FLIM

**Figure 5:**
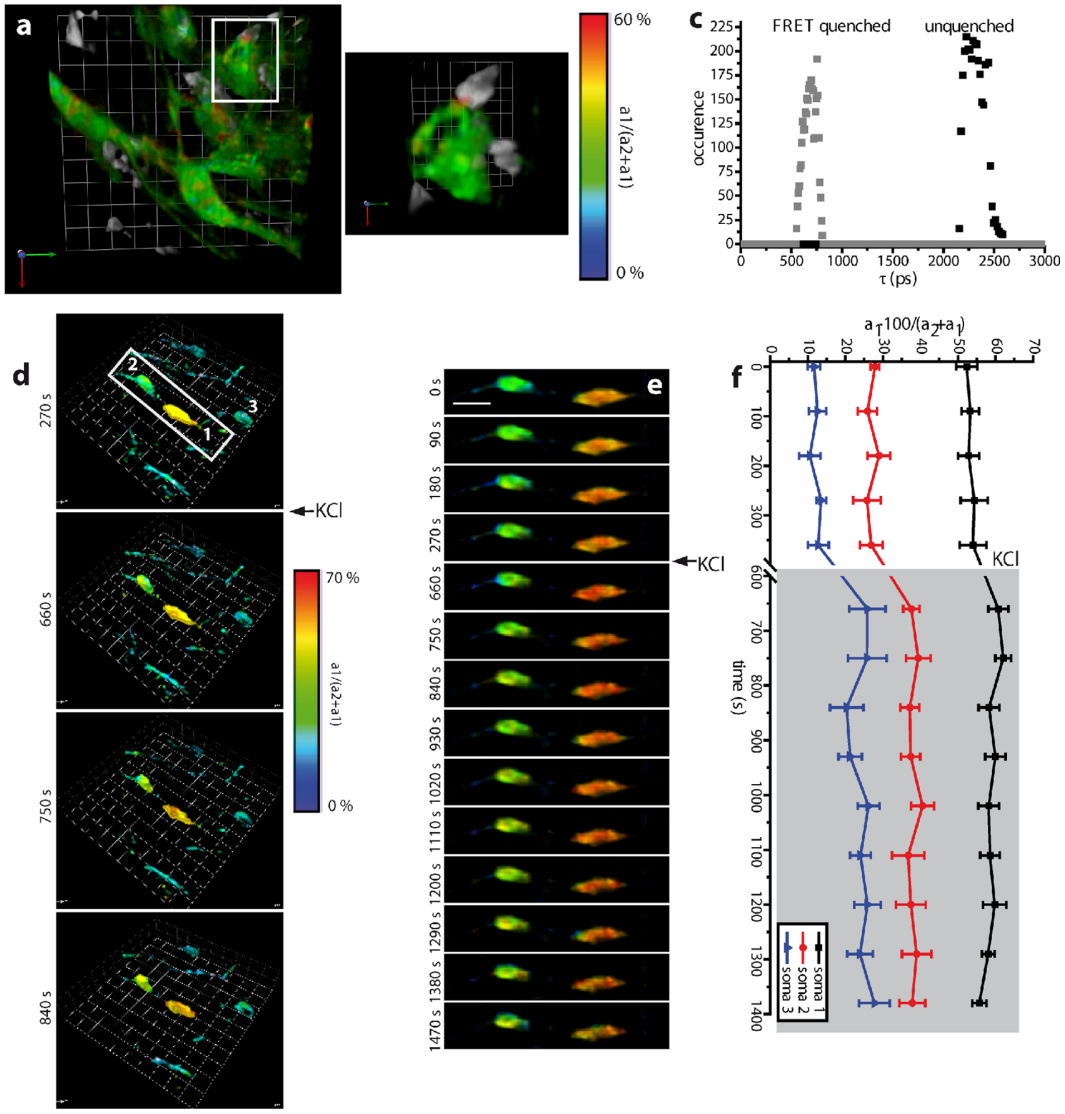
Dynamic intravital FRET-FLIM in the brain stem of *CerTN L15* mice. **(a)** Merged 3D a_1_·100/(a_2_+a_1_) image (FRET signal image, 150×150×30 µm^3^, 256×256×16 voxel in 90 µm depth) showing neuronal calcium and 3D data set of the tdRFP expressing immune cells recorded by the p-TCSPC setup. **(b)** As shown in the demarcated area in **(a)**, the calcium level in somata and processes interacting with immune cells, directly at the contact site, is significantly higher (FRET signal of up to 58%, 1.66 µM calcium, orange-red area in **(b)**) than in unaffected somata and processes (FRET signal of approximately 8%, 110 nM calcium). The corresponding fluorescence lifetime distributions of the FRET-quenched and unquenched Cerulean, as shown in the graph **(c)**, are similar to those determined in hippocampal slices. **(d)** The perfusion with a 100 mM KCl solution led to the depolarization of the neurons followed by a calcium concentration increase. The effect is more prominent in cells with low calcium than in the already affected neurons. **(e)** Time lapse of 3D a_1_·100/(a_2_+a_1_) images of two neurons in contact with immune cells and then being subject to K^+^ ion increase. The graph **(f)** shows the absolute values of the FRET signal in the somata numbered from 1 to 3 in **(d)**. 3D unit  = 15 µm, scale bar  = 12 µm. Imaging was performed between 80 µm to 100 µm depth in tissue, at a site with high immune cell infiltration grade (*Material S1*).

Using the p-TCSPC setup we were able to perform time-lapse 3D FLIM in 30 to 150 µm depth in the brain stem of live anesthetized *CerTN L15* chimeric mice which were reconstituted with a tdRFP expressing immune system. These mice were affected by experimental autoimmune encephalomyelitis (EAE) (Fig. 5). We were therefore able to quantify the absolute sub-cellular calcium concentration in neurons during direct interaction with immune cells. The calcium levels in neurons not in contact with immune cells was low, i.e. a_1_·100/(a_2_+a_1_) of 8±3% (227±27 nM calcium, n = 8, 5 mice, 2 different EAE cohorts), while in somata that were in contact with immune cells, the local calcium level increased up to 58% (1.66 µM calcium) (Fig. 5a, b). Our dynamic intravital FRET-FLIM experiments quantitatively validate for the first time our prior qualitative observation that sustained dynamic interaction between immune cells and neuronal structures in EAE (> 5 min) correlates with increased neuronal calcium levels at the contact site, which is followed by partially reversible neuronal dysfunction (> 1 µM calcium) as defined in [3].

Furthermore, the perfusion of the brain stem with 100 mM KCl solution lead to an increase of neuronal calcium in all neurons via membrane depolarization (Fig. 5d, e, f, *movie S3*). In low-calcium somata not in contact with immune cells, the calcium increase was more prominent than in high-calcium somata (Fig. 5e, f). Interestingly, the expected maximum calcium concentration (FRET signal 70%, 3.1 µM calcium) by depolarization is not reached. A possible explanation is the fact that in the living organism with intact blood flow, the locally applied KCl solution can be easily removed.

For these experiments, 3D time-resolved data sets of 150×150×30 µm^3^ (256×256×16 voxel) or of 150×150×12 µm^3^ (256×256×7 voxel) were acquired every 90 or 60 seconds (*Material S1*). The fluorescence lifetime of unquenched and FRET-quenched Cerulean, respectively, amounted to 2318 ps (217 ps FWHM) and to 686 ps (142 ps FWHM) (Fig. 5c) resulting from the non-linear bi-exponential evaluation (*Material S1*).

### Photobleaching and photodamage

The fluorescence signal loss due to fluorophore photobleaching and the functionality and morphology loss due to tissue photodamage are limiting factors in time-lapse deep-tissue intravital microscopy in general, and in time-lapse deep-tissue intravital FLIM, in particular.

The photobleaching of Cerulean present in the neurons of *CerTN L15* mice during repeated p-TCSPC imaging (*Fig. S5*) over 30 minutes (60 scans and 20 3D FLIM-images) was similar to that measured by standard TPLSM [35] under similar experimental conditions (*Material S1*).

Using the p-TCSPC setup we could perform FLIM over at least 2 hours in the brain stem of anesthetized healthy *CerTN L15* mice without observing any changes in the cellular calcium level (data not shown). Since the calcium level is a sensitive indicator of neuronal vital function [50], we conclude that p-TCSPC FLIM does not induce functionally discernible tissue photodamage.

## Discussion

Although two-photon laser-scanning microscopy (TPLSM) is the most powerful tool for high-resolution intravital imaging available to date [2]–[5], [51], it is limited in describing cellular function at the molecular level. Therefore, dynamic intravital fluorescence lifetime imaging (FLIM) is required [52]–[53]. Here we present a novel parallelized TCSPC (p-TCSPC) device featuring a 80 MHz average count rate, enabling us to demonstrate 4D-FLIM *in vivo*. Hence, p-TCSPC retains the optical performance and the accuracy of standard TCSPC techniques simultaneously allowing for dynamic FLIM over large regions (3D-stacks). As compared to video-rate FLIM based on gated optical intensifiers (GOI) [23] in the context of deep-tissue imaging, p-TCSPC shows, as expected, better optical and FLIM performance at a similar acquisition speed.

Our benchmarking experiments show that at high signals, exceeding single photon counting, multifocal GOI-FLIM is considerably faster than p-TCSPC-FLIM. Yet, at the low fluorescence signal intensity which is typical for deep-tissue imaging, this advantage vanishes. Decreasing the signal-to-noise ratio (SNR) leads to faster acquisition with both detectors, but also to lower FLIM accuracy. the advantage of p-TCSPC in acquisition speed over standard single-channel TCSPC devices is preserved as long as more than one photon every 8th laser pulse impinges on the photocathode, i.e. more 10^6^ photons are emitted per second. As demonstrated by us, this is the case even in dynamic intravital FLIM in more than 100 µm depth withinthe spinal cord of *CerTN L15* mice. The use of hybrid point-detectors (PMT-APD) with shorter transit times and improved detection efficiency (70% at 550 nm) in combination with single-channel electronics (10 MHz), is not sufficient to perform ultrafast FLIM. The use of hybrid detectors together with parallelized electronics will solve the mentioned limitations and will constitute a break-through in fast and ultra-fast deep-tissue FLIM.

As expected, both single-beam scanning TCSPC setups perform better than the multifocal GOI setup as far as depth-dependent spatial resolution and signal-to-noise ratio are concerned. Interestingly, we showed that the maximum imaging depth in brain and skin tissue determined by the depth-dependent SNR is larger than that of reasonable FLIM accuracy (FWHM  = 1000 ps). However, both values are up to three times larger for the TCSPC setups than for the GOI setup. Moreover, since the FLIM accuracy of the TCSPC devices, as PMT-based point-detectors, is limited only by the loss of excitation photons due to scattering, an exponential increase of the excitation power leads to increased FLIM depth in tissue. In contrast, the maximum FLIM depth of the GOI as a field-detector is mainly limited by the scattering of emitted photons. Thus, an excitation power increase would lead to larger ddSNR imaging depths, but would not allow for improvements of FLIM accuracy at deeper tissue layers.

Using the novel p-TCSPC device, we were for the first time able to precisely quantify neuronal calcium fluctuations as a read-out of neuronal dysfunction over time in response to chronic inflammation in the CNS of living *CerTN L15* mice affected by EAE.

In the context of rapid developments in transgenic mouse technology which aim to probe cellular, tissue and organ function at the molecular level *in vivo*, the development of fast, non-invasive and highly spatially and time-resolved FLIM represents a critical step. The parallelized TCSPC technique presented here allows for dynamic intravital FLIM in whole organs, and thus opens new perspectives in the investigation of cellular fate in genuine tissue environment.

## Supporting Information

Figure S1 **Experimental multi-photon setup for live tissue fluorescence lifetime imaging.** (**a**) Set up of the multi-photon microscope used in FLIM experiments. A Ti:Sa laser and an optical parametric oscillator (OPO) are used as excitation sources for multi-photon microscopy. Their beams are separately shaped. The Ti:Sa beam is either first split by the beam multiplexer in up to 64 beamlets (multifocal scanning mode) or simply directed to the galvanometric scanner. The spatial overlap of the Ti:Sa and OPO beams is achieved by a customized dichroic mirror (DM1) before the galvanometric scanner. The beams are then directed through a system of scan (SL) and tubus lenses (TL), through a dichroic mirror (DM2), finally to the objective lens. The objective lens focuses the excitation beams into the sample and collects the fluorescence. The fluorescence light is reflected by the dichroic mirror DM3 to the parallelized TCSPC detector or transmitted through DM2 and a near-infrared (NIR) blocking filter either to the gated optical intensifier (FLIM field-detector) or to standard photomultiplier tubes (PMT). (**b**) Working principle of the parallelized TCSPC. The fluorescence photons are led by a liquid light guide to the detector, a 16-anode PMT. The signal is homogenized prior to detection. The electronic signal of the PMTs is shaped by analog electronics. The events are counted by two groups of 8 time-to-digital converters (TDC). The photon counting information from the TDCs is converted to the final histogram by a FPGA module. (**c**) Typical fluorescence decays measured with the GOI setup at an arbitrary pixel of a 100×100 pixel image within a 4∶1 mixture of Rhodamine B and Rhodamine 6G (10 µM, aqueous solution) and fitted with a biexponential function (Levenberg-Marquadt algorithm). (**d**) Typical fluorescence decays measured with the p-TCSPC setup at an arbitrary pixel of a 100×100 pixel image within the same solution and fitted with the same algorithm. For both (c) and (d) the fitted parameters and the quality of the fit (χ^2^_R_) are given in the inset. (**e**) Fluorescence lifetime distributions of RhB and Rh6G corresponding to (c) over the 100×100 pixel image. (**f**) Fluorescence lifetime distributions of RhB and Rh6G corresponding to (d) over the 100×100 pixel image. The insets in the graphs (e) and (f) are intensity images of similar signal-to-noise ratio immediately after excitation, at the onset of fluorescence.
(PDF)

Figure S2 **Benchmarking accuracy and acquisition speed in FLIM. (a)** and **(b)**: Examples of typical monoexponential fluorescence decays and fitting curves of a pixel in 10 µM Rhodamin 6G aqueous solution acquired by the GOI setup and by the p-TCSPC setup, respectively. The insets depict the fitted parameters and the quality of the fit (χ^2^_R_). The graphs **(c)** and **(e)** show the 400 ps time-gate of the GOI detector and the instrument response function of the p-TCSPC detector, respectively. Fluorescence lifetime distributions of 100×100 pixel images of similar SNR corresponding to the decays depicted in (a) and (b) are shown in **(d)** and **(f).** The graphs (**g**) and (**h**) depict typical monoexponential decays (100×100 pixel images of similar SNR) measured by the GOI and p-TCSPC setup, respectively, in a 10 µM solution of Rhodamin B. The fitted parameters and χ^2^_R_ are shown in the insets. (**i**) and (**j**): Typical biexponential fluorescence decays and fitting curves of one pixel in a 1∶1 mixture of 10 µM Rhodamin B and 10 µM Rhodamin 6G aqueous solutions acquired by the GOI setup and by the p-TCSPC setup, respectively. All setup parameters are listed in *Material S1.*
(PDF)

Figure S3 **Spatial resolution in FLIM.** Lateral and axial profiles (**a**) and xz cross-section (**b**) of ePSF of fluorescing 200 nm beads embedded in agarose measured by the GOI and p-TCSPC detector, respectively. λ_exc_  = 800 nm, z step-size  = 200 nm, λ_detection_  = 525±25 nm. (**c**) Fluorescence images of the same region of a skin biopsy stained with FITC acquired in 20 and in 60 µm depth with the GOI and p-TCSPC detector, respectively. In 60 µm depth in skin tissue the p-TCSPC setup still depicts sub-cellular details, while the GOI setup sparsely restores the morphology. The FLIM image acquisition time and excitation power was similar for both setups at λ_exc_  = 770 nm and λ_detection_  = 525±25 nm (*Material S1, Supplemental Setup Parameters*).
(PDF)

Figure S4 **Typical evaluation algorithms using the Becker&Hickl FLIM-software for the FRET-FLIM data measured with the single-channel TCSPC based on a hybrid detector (Becker&Hickl).** The time-resolved fluorescence of Cerulean in the spinal cord of healthy CerTN L15 mice is acquired at 850 nm so that no more than 10^6^ photons/s are evaluated. This is required to avoid pile-up effects. Both iterative biexponential approximations by means of Levenberg-Marquardt algorithms and bilinear regressions with fixed fluorescence lifetimes of unquenched and FRET-quenched Cerulean are applied.
(PDF)

Figure S5 **Photobleaching in dynamic intravital p-TCSPC FLIM. (a)** Time-lapse of 3D Cerulean fluorescence images of neuronal processes in the brain stem of a *CerTN L15* mouse as acquired by p-TCSPC FLIM. λ_exc_  = 850 nm, z step-size  = 2 µm, λ_detection_  = 475±20 nm, peak laser power 3.13·10^5^ mW. **(b)** Corresponding loss of the normalized Cerulean fluorescence over time, i.e. number of scans, due to photobleaching.
(PDF)

Movie S1 **Neuronal response to KCl in hippocampus slices of** ***CerTN L15*** **mice measured by GOI-based FLIM.** 3D movie of a_1_·100/(a_2_+a_1_) images (FRET signal, Calcium level) in hippocampal slices of a *CerTN L15* mouse before (first image) and during perfusion with 100 mM KCl. The acquisition was performed with the GOI-based FLIM setup in the 16 beam scanning mode (*Material S1, Supplemental Setup Parameters*). Scale bar  = 20 µm.
(ZIP)

Movie S2 **Neuronal response to KCl in hippocampus slices of** ***CerTN L15*** **mice measured by p-TCSPC FLIM.** 3D movie of a_1_·100/(a_2_+a_1_) images (FRET signal, Calcium level) in hippocampal slices of a *CerTN L15* mouse before (first image) and during perfusion with 100 mM KCl. The acquisition was performed with the p-TCSPC FLIM setup in the single beam scanning mode (see *Material S1*). Scale bar  = 20 µm.
(ZIP)

Movie S3 **Neuronal response to direct interaction with immune cells in the brain stem of** ***CerTN L15*** **mice affected by EAE, measured by p-TCSPC FLIM.** 3D movie of a_1_·100/(a_2_+a_1_) images (FRET signal, Calcium level) in the brain stem of a *CerTN L15* mouse affected by experimental autoimmune encephalomyelitis before (first five images) and during local perfusion with 100 mM KCl. The acquisition was performed with the p-TCSPC FLIM setup in the single beam scanning mode (see *Material S1*). Scale bar  = 20 µm.
(ZIP)

Material S1 **In the additional Supplemental Material and Methods we describe in more detail the technical setup of the microscope, working principles of compared detectors used in experiments and sample preparation.** Detailed **Supplemental Setup Parameters** regarding the FLIM benchmarking experiments, PSF, ddSNR and dynamic intravital imaging experiments are included. **Supplemental data analysis** describes the algorithms and software used for data analysis. Further relevant literature is included in Supplemental References**.**
(DOCX)
