# Supplementary figures and images for: Parallelized TCSPC for Dynamic Intravital Fluorescence Lifetime Imaging: Quantifying Neuronal Dysfunction in Neuroinflammation
Source: PLoS One. 2013 Apr 16;8(4):e60100. doi: 10.1371/journal.pone.0060100 (PMC3629055; doi:10.1371/journal.pone.0060100)

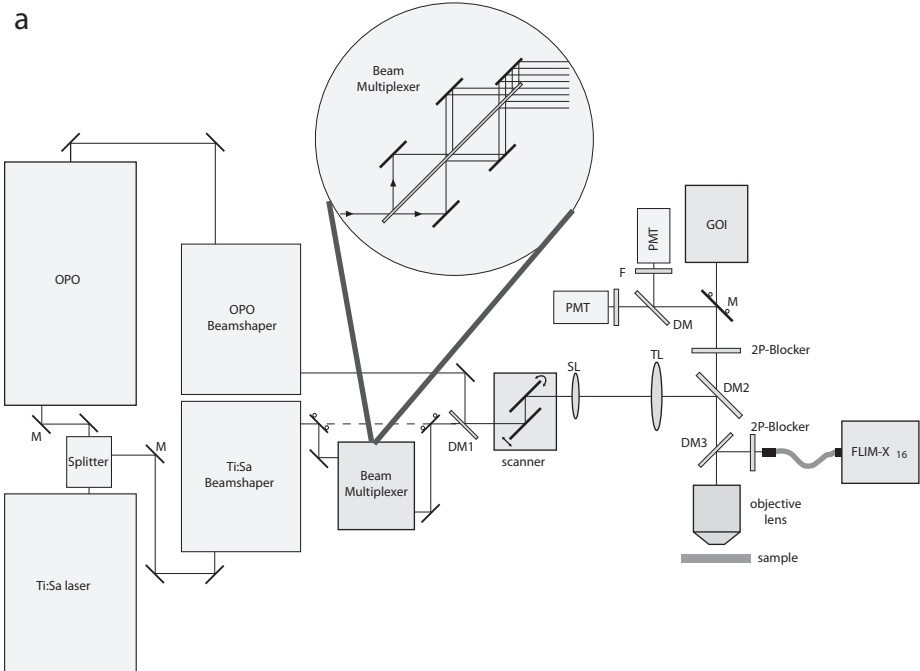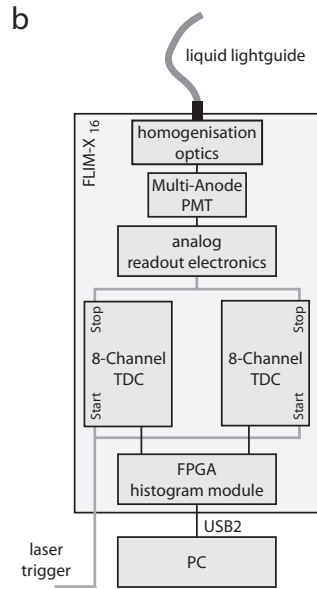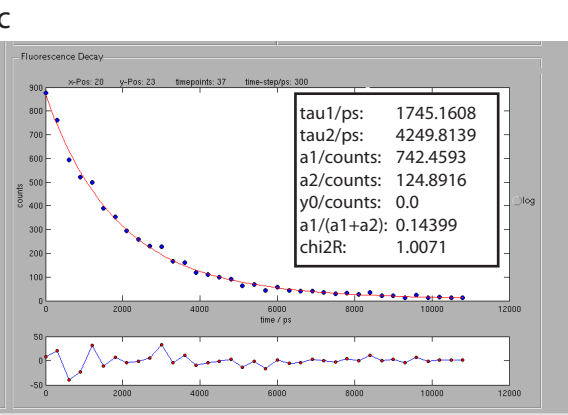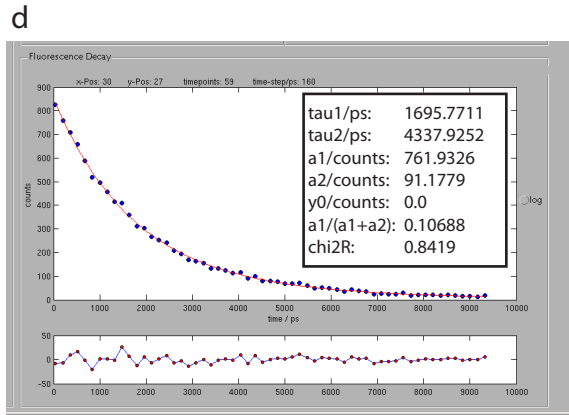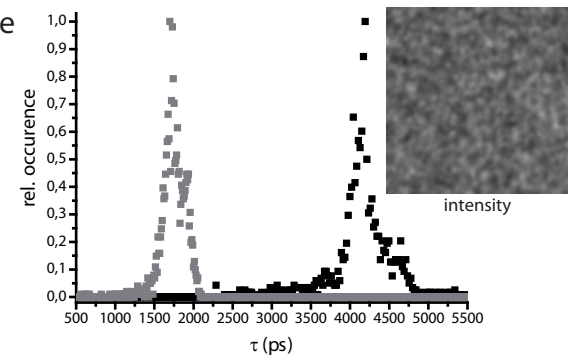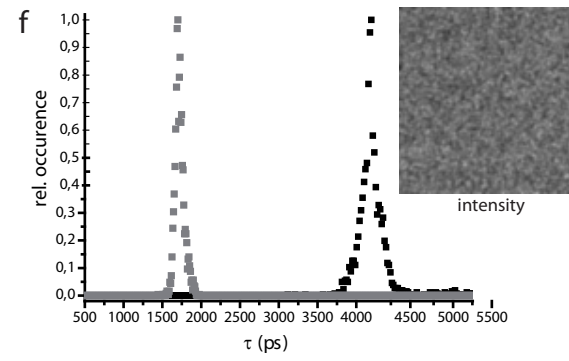

Supplement: Figure S1 — Experimental multi-photon setup for live tissue fluorescence lifetime imaging. (a) Set up of the multi-photon microscope used in FLIM experiments. A Ti:Sa laser and an optical parametric oscillator (OPO) are used as excitation sources for multi-photon microscopy. Their beams are separately shaped. The Ti:Sa beam is either first split by the beam multiplexer in up to 64 beamlets (multifocal scanning mode) or simply directed to the galvanometric scanner. The spatial overlap of the Ti:Sa and OPO beams is achieved by a customized dichroic mirror (DM1) before the galvanometric scanner. The beams are then directed through a system of scan (SL) and tubus lenses (TL), through a dichroic mirror (DM2), finally to the objective lens. The objective lens focuses the excitation beams into the sample and collects the fluorescence. The fluorescence light is reflected by the dichroic mirror DM3 to the parallelized TCSPC detector or transmitted through DM2 and a near-infrared (NIR) blocking filter either to the gated optical intensifier (FLIM field-detector) or to standard photomultiplier tubes (PMT). (b) Working principle of the parallelized TCSPC. The fluorescence photons are led by a liquid light guide to the detector, a 16-anode PMT. The signal is homogenized prior to detection. The electronic signal of the PMTs is shaped by analog electronics. The events are counted by two groups of 8 time-to-digital converters (TDC). The photon counting information from the TDCs is converted to the final histogram by a FPGA module. (c) Typical fluorescence decays measured with the GOI setup at an arbitrary pixel of a 100×100 pixel image within a 4∶1 mixture of Rhodamine B and Rhodamine 6G (10 µM, aqueous solution) and fitted with a biexponential function (Levenberg-Marquadt algorithm). (d) Typical fluorescence decays measured with the p-TCSPC setup at an arbitrary pixel of a 100×100 pixel image within the same solution and fitted with the same algorithm. For both (c) and (d) the fitted paramete [file pone.0060100.s001.pdf]

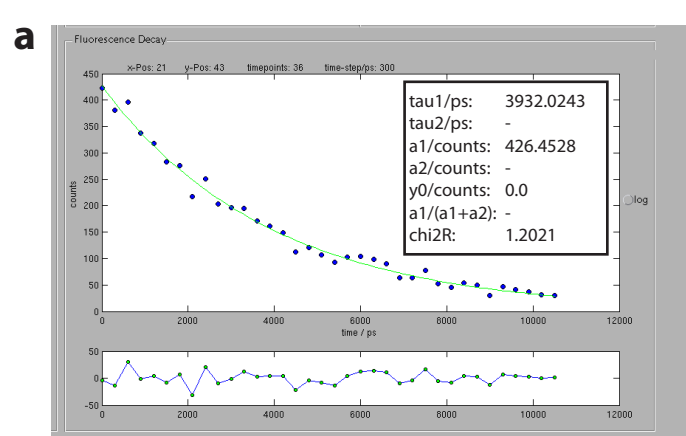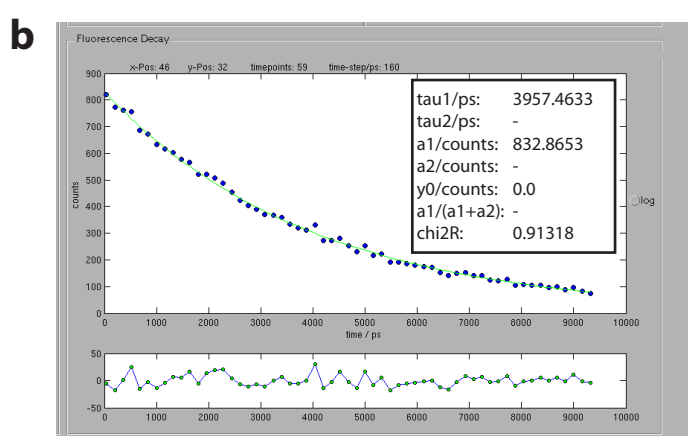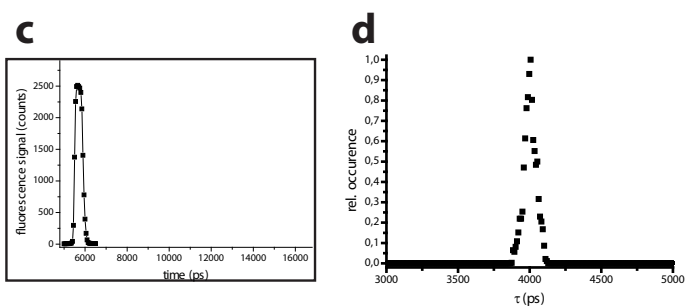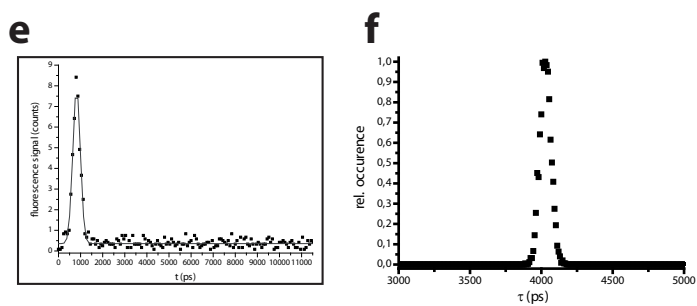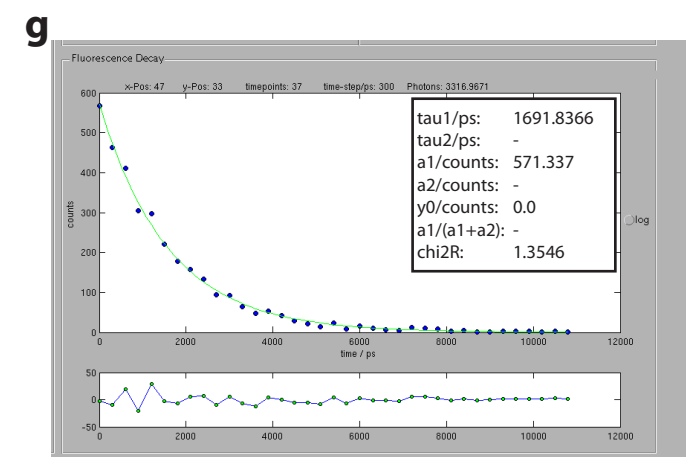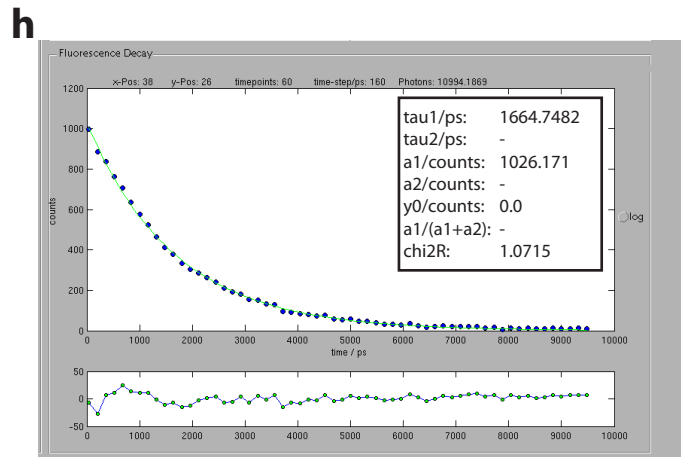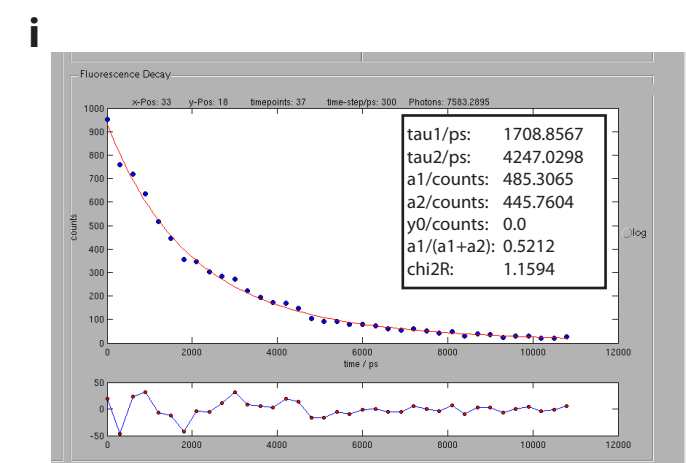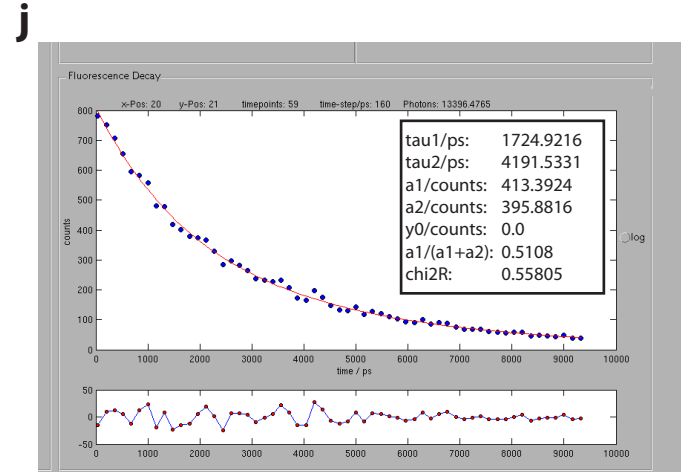

Supplement: Figure S2 — Benchmarking accuracy and acquisition speed in FLIM. (a) and (b): Examples of typical monoexponential fluorescence decays and fitting curves of a pixel in 10 µM Rhodamin 6G aqueous solution acquired by the GOI setup and by the p-TCSPC setup, respectively. The insets depict the fitted parameters and the quality of the fit (χ2 R). The graphs (c) and (e) show the 400 ps time-gate of the GOI detector and the instrument response function of the p-TCSPC detector, respectively. Fluorescence lifetime distributions of 100×100 pixel images of similar SNR corresponding to the decays depicted in (a) and (b) are shown in (d) and (f). The graphs (g) and (h) depict typical monoexponential decays (100×100 pixel images of similar SNR) measured by the GOI and p-TCSPC setup, respectively, in a 10 µM solution of Rhodamin B. The fitted parameters and χ2 R are shown in the insets. (i) and (j): Typical biexponential fluorescence decays and fitting curves of one pixel in a 1∶1 mixture of 10 µM Rhodamin B and 10 µM Rhodamin 6G aqueous solutions acquired by the GOI setup and by the p-TCSPC setup, respectively. All setup parameters are listed in Material S1. (PDF) [file pone.0060100.s002.pdf]

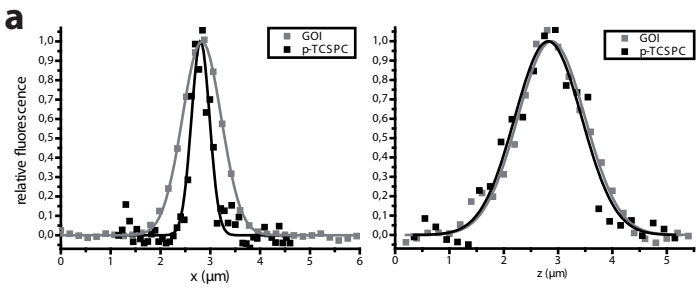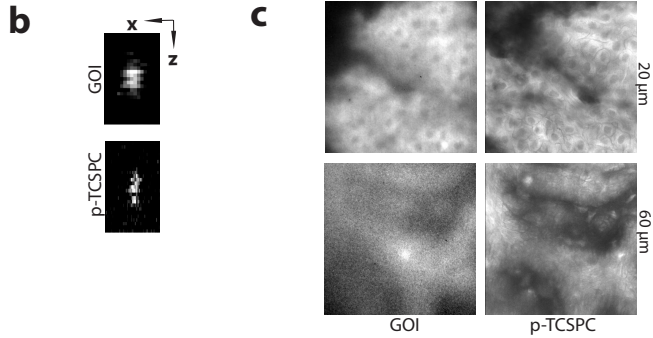

Supplement: Figure S3 — Spatial resolution in FLIM. Lateral and axial profiles (a) and xz cross-section (b) of ePSF of fluorescing 200 nm beads embedded in agarose measured by the GOI and p-TCSPC detector, respectively. λexc = 800 nm, z step-size = 200 nm, λdetection = 525±25 nm. (c) Fluorescence images of the same region of a skin biopsy stained with FITC acquired in 20 and in 60 µm depth with the GOI and p-TCSPC detector, respectively. In 60 µm depth in skin tissue the p-TCSPC setup still depicts sub-cellular details, while the GOI setup sparsely restores the morphology. The FLIM image acquisition time and excitation power was similar for both setups at λexc = 770 nm and λdetection = 525±25 nm (Material S1, Supplemental Setup Parameters). (PDF) [file pone.0060100.s003.pdf]

**a**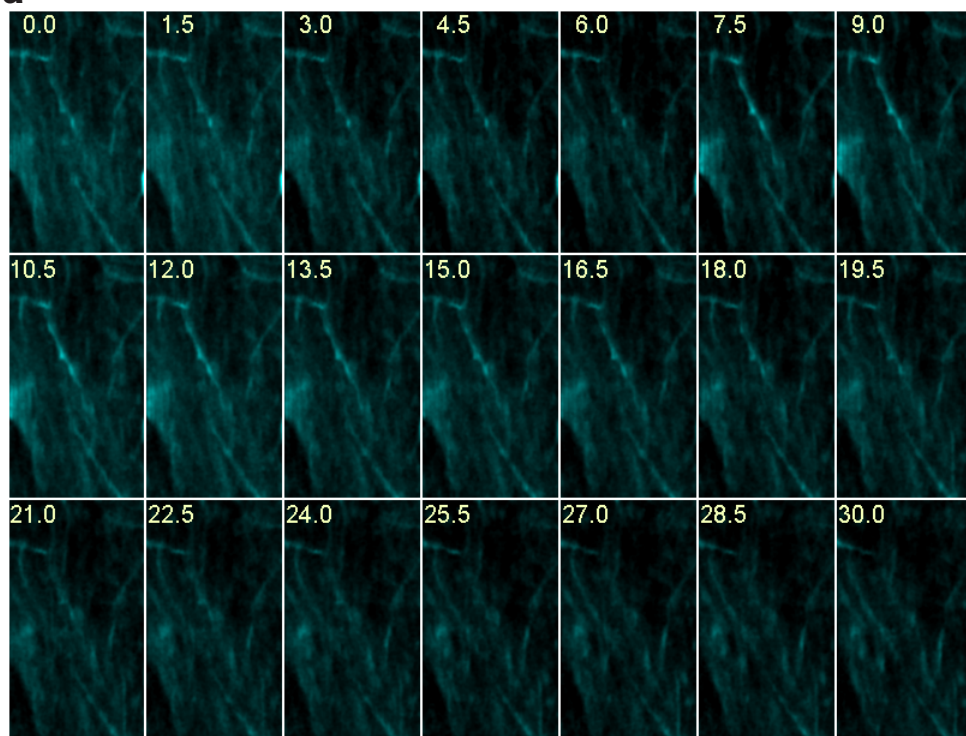**b**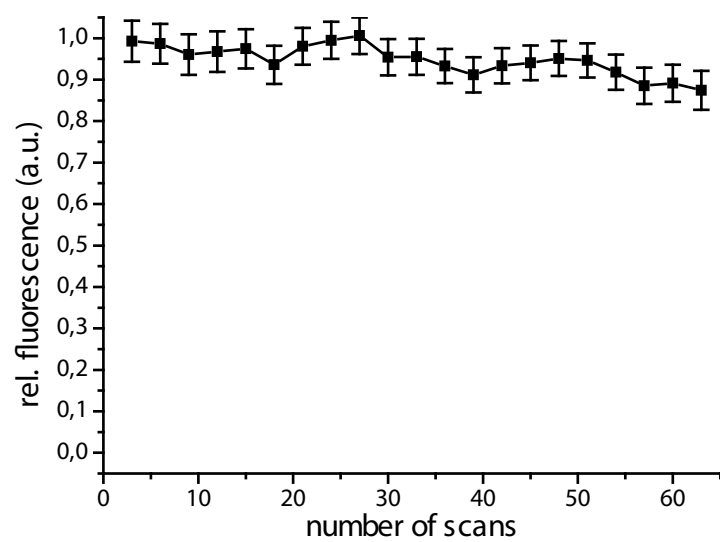

Supplement: Figure S5 — Photobleaching in dynamic intravital p-TCSPC FLIM. (a) Time-lapse of 3D Cerulean fluorescence images of neuronal processes in the brain stem of a CerTN L15 mouse as acquired by p-TCSPC FLIM. λexc = 850 nm, z step-size = 2 µm, λdetection = 475±20 nm, peak laser power 3.13·105 mW. (b) Corresponding loss of the normalized Cerulean fluorescence over time, i.e. number of scans, due to photobleaching. (PDF) [file pone.0060100.s005.pdf]
