# Supplementary material for: Parallelized TCSPC for Dynamic Intravital Fluorescence Lifetime Imaging: Quantifying Neuronal Dysfunction in Neuroinflammation
Source: PLoS One. 2013 Apr 16;8(4):e60100. doi: 10.1371/journal.pone.0060100 (PMC3629055; doi:10.1371/journal.pone.0060100)

Biexponential evaluation of the FRET signal in CerTN L15 mice (Levenberg-Marquardt)

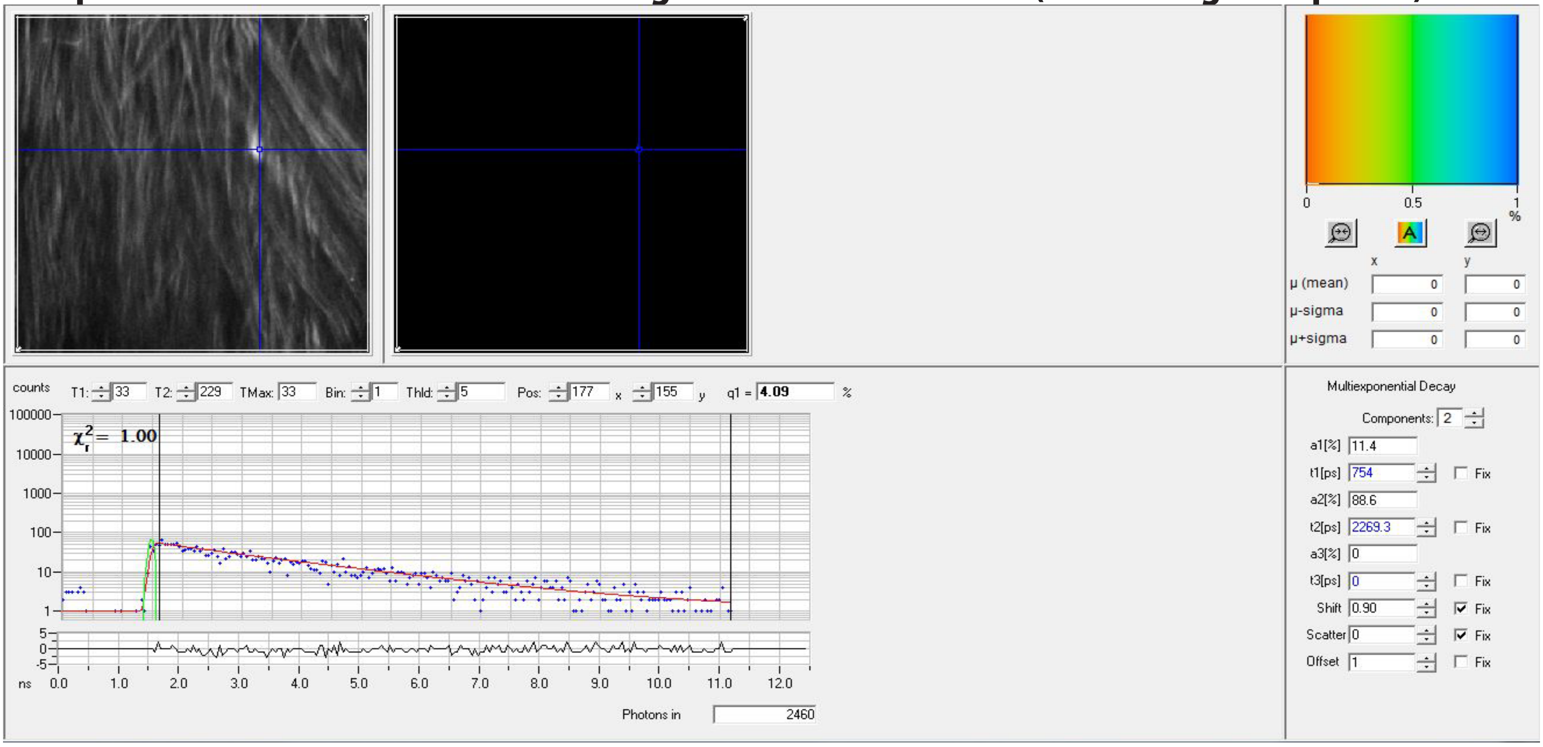

Bilinear regressive evaluation of the FRET signal in CerTN L15 mice

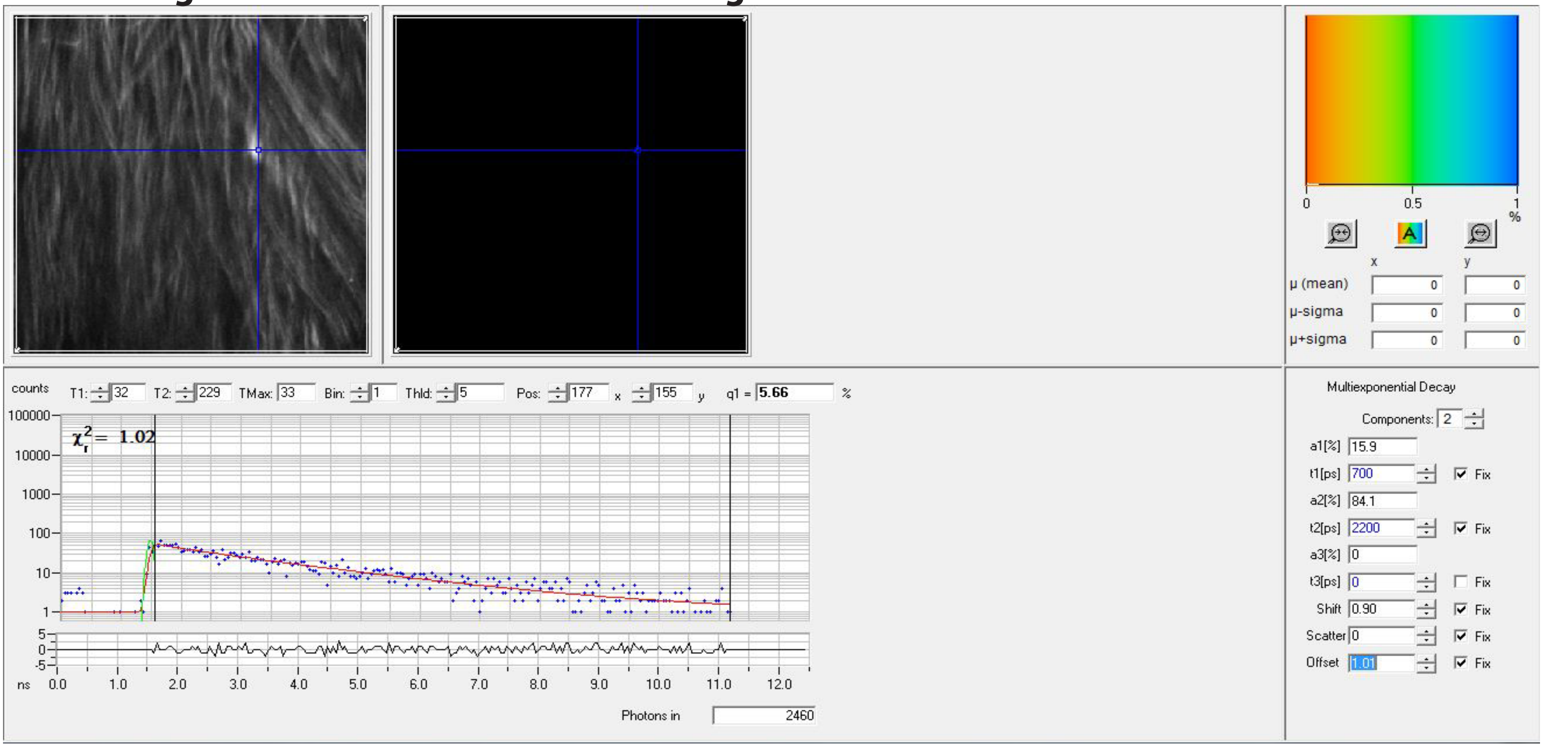

Supplement: Figure S4 — Typical evaluation algorithms using the Becker&Hickl FLIM-software for the FRET-FLIM data measured with the single-channel TCSPC based on a hybrid detector (Becker&Hickl). The time-resolved fluorescence of Cerulean in the spinal cord of healthy CerTN L15 mice is acquired at 850 nm so that no more than 106 photons/s are evaluated. This is required to avoid pile-up effects. Both iterative biexponential approximations by means of Levenberg-Marquardt algorithms and bilinear regressions with fixed fluorescence lifetimes of unquenched and FRET-quenched Cerulean are applied. (PDF) [file pone.0060100.s004.pdf]
