# Supplementary material for: Parallelized TCSPC for Dynamic Intravital Fluorescence Lifetime Imaging: Quantifying Neuronal Dysfunction in Neuroinflammation
Source: PLoS One. 2013 Apr 16;8(4):e60100. doi: 10.1371/journal.pone.0060100 (PMC3629055; doi:10.1371/journal.pone.0060100)
Supplement: Material S1 — In the additional Supplemental Material and Methods we describe in more detail the technical setup of the microscope, working principles of compared detectors used in experiments and sample preparation. Detailed Supplemental Setup Parameters regarding the FLIM benchmarking experiments, PSF, ddSNR and dynamic intravital imaging experiments are included. Supplemental data analysis describes the algorithms and software used for data analysis. Further relevant literature is included in Supplemental References. (DOCX) [file pone.0060100.s009.docx]

**Supplemental Material S1: Parallelized TCSPC for dynamic intravital fluorescence lifetime imaging: quantifying neuronal dysfunction in neuroinflammation**

Jan Leo Rinnenthal4,1§, Christian Börnchen3§, Helena Radbruch4,2, Volker Andresen5, Volker Siffrin7,2, Thomas Seelemann6, Heinrich Spiecker5, Agata Mossakowski4,1, Ingrid Moll3, Josephine Herz2,4,#, Anja E. Hauser1, Frauke Zipp7,2, Martin J. Behne3* and Raluca Niesner1,2,4*

*1 German Rheumatism Research Center, Berlin, Germany*

*2 Max-Delbrück Center for Molecular Medicine, Berlin Germany*

*3 Department of Dermatology and Venerology, University Medical Center Hamburg-Eppendorf, Hamburg, Germany*

*4 Charité – University of Medicine, Berlin Germany*

*5 LaVision Biotec GmbH, Bielefeld, Germany*

*6 LaVision, Göttingen, Germany*

*7 Neurology, Johannes Gutenberg University Mainz,* *Mainz, Germany*

*#current affiliation: Neurology department, University Duisburg-Essen, Essen, Germany*

*§* equally contributing § first and *senior authors*

**Supplemental Material**

1. **Supplemental material and methods**
2. **Supplemental setup parameters**
3. **Supplemental data analysis**

**I. Supplemental material and methods**

**Two-photon laser-scanning microscope (TPLSM)**

Experiments were performed using a specialized multi-photon laser-scanning microscope (*Fig. S1a*) for fluorescence lifetime imaging (FLIM) [[1](#_ENREF_1),[2](#_ENREF_2)] In brief, the beam of a tunable fs-pulsed Ti:Sa laser (wavelength range 700 – 1020 nm, 100 fs, 80 MHz, MaiTai, Spectra Physics, Germany) is either directly scanned by two galvanometric mirrors or split into 2, 4, 8 up to 64 beams before reaching the scanning mirrors. Two neighbouring beamlets have perpendicular polarizations and are time shifted. The time shift between the first and the 64th beamlet amounts to 3 ps. For image acquisition, the single beam or the variable beamlet line is focused into the sample by an objective lens for deep-tissue imaging (20x dipping lens, NA 0.95, WD 2 mm – Olympus, Hamburg, Germany) and scanned over the sample. The fluorescence signal is detected and analyzed either by a time-gated MCP-based field detector in the multi-beam scanning mode (GOI detector PicoStar, LaVision, Göttingen, Germany, combined with the CCD camera PixelFly QE, PCO, UK) or by one of the two TCSPC detectors in the single-beam scan-mode: the novel parallelized 16 channel TCSPC point detector (p-TCSPC FLIM-X_16_, LaVision Biotec GmbH, Bielefeld) or a single-channel TCSPC system combining a hybrid detector HPM-100-40 with the SPC-150 counting electronics (average rate 10 MHz) (Becker&Hickl GmbH, Berlin, Germany). The p-TCSPC device is based on parallel photon detection with multi-anode (16 channels) photomultiplier tubes (PMT) and on evaluation relying on time-to-digital converter (TDC) electronics. Thus, the electronic dead time of the device is reduced to 5.5 ns (best values for TCSPC devices are currently approx. 80 ns [[3](#_ENREF_3)]) while the FLIM repetition rate is limited only by the laser, i.e. 80 MHz, and no longer by the TCSPC electronics. Spectral discrimination of the fluorescence signal was achieved by appropriate dichroic mirrors and interference filters. Rhodamin 6G and Rhodamin B were excited at 800 nm and detected above 560 nm. FITC was excited at 770 nm and detected at 525 ± 25 nm. EGFP expressed in neurons of *Thy1 EGFP* mice was excited at 850 nm and detected through an interference filter at 525 ± 25 nm. Cerulean (CFP derivative) in the neurons of *CerTN L15* mice was excited at 850 nm and detected through a dichroic mirror (cut off wavelength 506 nm) and an interference filter of 475 ± 20 nm.

In intravital FRET-FLIM experiments on *CerTN L15* chimera mice reconstituted with a tdRFP expressing immune system we employed dual near infrared/infrared excitation. Therefore, additional to the Ti:Sa laser tuned to 850 nm, we used an optical parametric oscillator (OPO, APE, Berlin, Germany) tuned to 1110 nm [4].

**Parallelized TCSPC: working principle**

The novel TCSPC detector is a parallelized photon counting device (p-TCSPC) consisting of a 16-anode PMT, followed by analog electronics and two groups of 8 TDC-based single-photon counting electronics with a time bin of 80 ps (*Fig. S1b*). In front of the PMT, diffusion optics is used to spread photons originating from a single spot within the sample homogenously across the photocathode. The used 16-anode PMT is characterized by a quantum efficiency of 18% at 530 nm. Each resulting photoelectron is amplified by one of the 16 channels of electrodes (800 V applied voltage on each channel, gain 10^6^) and detected by one of the 16 anodes. Hence, up to 16 photons can be detected and processed in parallel. Although the detection efficiency of the 16 anode PMT is lower than that of other point detectors, e.g. characterized by up to 45% quantum efficiency, the parallelization of detection and signal amplification dramatically improves the photon economy as compared to standard PMTs by reducing the apparent transit time through the detector. The constantly incoming photoelectrons are homogenously distributed on two groups of 8 TDCs-based electronics. In front of the TDCs constant fraction discriminators (CFD) build the derivative of the single-event pulse (TTS 180 ps, rise time 600 ps). The TDCs are, as previously described [[2](#_ENREF_2)], piezoelectric crystals which accurately oscillate at a high frequency if a voltage is applied. Thus, they can be used as counters for the time period between the photon arrival and laser pulse arrival. The laser pulses train is detected by an ultrafast photodiode (instrument response function < 300 ps; OCF, Becker&Hickl, Berlin, Germany). The resulting pulse is amplified and transformed into a TTL pulse trigger by a trimmer box. This is derived by a CFD and simultaneously sent to the two groups of TDCs. Base-calibration via precise electronics within the FPGA unit enables synchronized registration of the photoelectron pulses on all 8 TDC channels within a group and between the two groups. The FPGA unit collects the photon arrival information of the two groups of TDCs and restores the single-photon counting histogram.

The device (and each TDC channel) features a dead time of only 5.5 ns and an average count rate of 80 MHz. An average count rate of 180 MHz / channel and a maximal count rate of 2 GHz are theoretically possible, but are limited by the laser repetition rate (80 MHz). The width of the instrument response function (IRF) as measured by SHG amounts to 280 ps. Further, the IRF is highly symmetric (no after pulsing) and can be well approximated by a Gaussian distribution (in *Fig. S1c*). The jitter of the instrument lays at <10 ps. The mean dark counts / channel amount to 5000 cps and do not exceed 10000 cps. The cross talk between TDC channels is 3%.

**Gated optical intensifier: working principle**

The principle of time-gated field-detection was previously described [[4](#_ENREF_4)]. In brief, the fluorescence photons impinge the photocathode of the gated optical intensifier (GOI). The photoelectrons are accelerated to the multi channel plate (MCP) only if a voltage is applied between the photocathode and MCP. A time-gate of 200 to 1000 ps is shifted to different positions with respect to the excitation pulse, thus generating the fluorescence decay curve. The repetition rate of the time-gate switch lays at 80 MHz. The photoelectrons amplified by the MCP are transformed by a phosphorus screen back into photons and then detected by a CCD camera. The detector is best suited for wide-field microscopy or for multifocal multi-photon microscopy to perform video-rate FLIM. We found that in order to achieve highest lifetime accuracy in the range 400 ps – 5000 ps a time-gate of 400 ps is optimal for best photon exploitation (data not shown). Up to date, the multifocal multi-photon GOI-based technique was the only compatible with dynamic fluorescence lifetime imaging in live tissue.

**Agarose gel**

For comparative point spread function PSF measurements, 200 nm fluorescent polystyrene beads (515 nm, Invitrogen, Germany) were embedded in a 2% agarose gel and placed on a glas slide for microscopy prior to imaging.

**Calcium calibration**

A homogenized cytosolic suspension containing the TN L15 construct (Troponin C bound to Cerulean and Citrine) was isolated from the lysate of fluorescing cells obtained after sorting the digested central nervous system of *CerTN L15* mice. The digestion was performed with collagenase and anti-myelin beads. The series of solutions of known calcium concentration was obtained using the Calium Calibration Kit (Invitrogen, Germany).

**Mice**

The *CerTN L15* mouse was descried elsewhere [[5](#_ENREF_5)]. Briefly, it expresses a FRET-based Ca-biosensor via *Thy1* expression cassette, e.g. in neurons. The Ca-biosensor *TN L15* is based on Troponin C bound to Cerulean (CFP derivative) and Citrine (YFP derivative) building the FRET pair. The *TN L15* sensor covers a dynamic range of Ca concentration from 100 nM to 10 µM, i.e. from the physiological to the pathological range. The *Thy1 EGFP* mouse [[6](#_ENREF_6)] (JAX Laboratory, USA) expresses EGFP in neurons. In C57BL/6 acRFPmice tdRFP (kindly provided by H. J. Fehling) is expressed under the Rosa26 locus [[7](#_ENREF_7)].

**Bone marrow chimera and experimental autoimmune encephalomyelitis (EAE)**

Bone marrow chimera mice were generated as previously described [[8](#_ENREF_8)]. In brief, recipient *CerTN L15* transgenic C57Bl/6 mice were sublethally irradiated and reconstituted immediately with CD90 depleted bone marrow cells from C57BL/6 acRF*P (“∆Neo-flip”)* donor mice. EAE was induced as previously described [[8](#_ENREF_8),[9](#_ENREF_9)]. Briefly, mice were immunized subcutaneously with 150 µg of MOG_35–55_ (Pepceuticals, UK) emulsified in CFA (BD Difco, Germany) and received 200 ng Pertussis toxin (PTx, List Biological Laboratories, Inc.) intraperitoneally at the time of immunization and 48 h later. Intravital multi-photon microscopy was performed at the peak of disease.

**Skin biopsy preparation and staining**

After cleaning and disinfection of pig ears, 6 mm punch biopsies were taken 1 h after slaughtering. The biopsies were placed dermis down on sterile gauze in culture dishes and immersed in medium, in such a way that only the dermis was in contact with the medium, whereas the epidermis remained exposed to air. The medium consisted of Dulbecco’s modified Eagle medium supplemented with hydrocortisone, 2% fetal calf serum, penicillin, and streptomycin. The biopsy was incubated with 10% CO_2_ at 37°C, under addition of a 100 µM fluorescein-isothio-cyanate (FITC) solution for 12 h prior to imaging.

**Preparation of the brain stem / spinal cord window for intravital imaging**

The brain stem was exposed by carefully removing the musculature above the dorsal neck area and removing the dura mater between the first cervical vertebra and the occipital skull bone. The head was inclined for access to deeper brainstem regions and the brain stem superfused with isotonic Ringer solution. Anesthesia depth was controlled by continuous CO_2_ measurements of exhaled gas and recorded with a CI-240 Microcapnograph (Columbus Instruments, USA) and by an Einthoven three-lead electrocardiogram (ECG). In order to avoid the strong breathing artefacts in the brainstem of anesthetized mice, the ECG signal was used as an external trigger for the image acquisition software, which controls the hardware of the microscope setup. Thus, each fluorescence stack was recorded at the same respiration state of the mouse and also in the same tissue region [[8](#_ENREF_8),[9](#_ENREF_9)].

In the case of intravital spinal cord imaging, a vertebra was removed in the lumbar region of the back bone to expose a region of approx. 2 x 1 mm² of the spinal cord. The region of interest within the spinal cord was fixed by two opposed forceps aligned along the back bone. The further preparation of the mouse for spinal cord intravital imaging is similar to that of the brain stem.

**II. Supplemental setup parameters**

**FLIM benchmarking experiments**

The setup parameters for the FLIM experiments on the 100 µM Rhodamin 6G water solution were: for the GOI setup - 64 beam scanning at 1200 Hz, peak photon flux *_Ø_* = 4.46·10^30^ photons/s·cm², time-gate 400 ps, gain 800V, two data points at 200 ps and 4500 ps after the laser pulse, exposure time = 15 ms, image size 150x150 µm² (215x215 pixel) and for the p-TCSPC setup - single beam scanning at 1200 Hz, peak photon flux at the sample *_Ø_* = 4.36·10^30^ photons/s·cm², time window 80 ps, 157 data points, gain 230 V, image size 150x150 µm² (512x512 pixel), multiple scanning = 1.

The measurement parameters for the mono- and biexponential FLIM experiments on diluted solutions were as follows: for the GOI setup – 32 beam scanning at 1200 Hz, peak photon flux *_Ø_* = 4.57·10^29^ photons/s·cm², time-gate of 400 ps, gain 800V, 20 to 40 data points on the fluorescence decay, exposure time = 44 ms or 87 ms, image size 33x33 µm² (100x100 pixel) and for the p-TCSPC setup - single beam scanning at 800 Hz or 1200 Hz, peak photon flux at the sample *_Ø_* = 4.14·10^29^ photons/s·cm², time window 80 ps or 160 ps, 157 or 78 data points, gain 230 V, image size 29x29 µm² (100x100 pixel), multiple scanning = 12 or 20. Evaluation was also performed in both cases with a non-linear Levenberg-Marquardt algorithm.

The experimental parameters of the single-channel electronics TCSPC setup were similar to that of the p-TCSPC setup except for the peak photon flux for which we choose a reduced value (*_Ø_* = 8.28·10^28^ photons/s·cm²) to avoid pile up.

The excitation wavelength λ_exc_ was 800 nm, the pulse width at the sample surface was 120 fs and detection was performed through a 593 ± 20 nm interference filter.

**ePSF measurements**

100 nm fluorescing beads (505/515) embedded in agarose were excited at 800 nm. Their fluorescence was detected through a 525 ± 25 nm interference filter. For the GOI setup we used 64 beam scanning at 400 Hz, peak photon flux *_Ø_* = 2.11·10^29^ photons/s·cm², time-gate 400 ps, gain 800V, 20 to 40 data points on the fluorescence decay, exposure time = 53.8 ms, image size 100x100 µm² (606x606 pixel). For the p-TCSPC setup we employed single beam scanning at 1200 Hz, peak photon flux at the sample *_Ø_* = 2.18·10^29^ photons/s·cm², time window 80 ps, 157 data points, gain 230 V, image size 150x150 µm² (512x512 pixel), multiple scanning = 1. 3D images were acquired with a step-size along the optical axis (z axis) of 300 nm.

The FITC stained skin biopsies were excited at 770 nm, with λ_detection_ = 525±25 nm, at peak laser power per beam of 2.9·10^5^ mW and 2.6·10^5^ mW for the GOI and p-TCSPC setup, respectively. 200x200 µm² images were acquired by 32-beam scanning at 1200 Hz in the GOI setup, time gate 400 ps, gain 800 V, exposure time = 261 ms and by single beam scanning at 1200 Hz in the p-TCSPC setup, gain 230 V, time window 80 ps, multiple scanning = 3.

**ddSNR and maximum FLIM depth**

3D time-resolved fluorescence images of FITC stained skin biopsies were acquired both with the GOI and the p-TCSPC setup at λ_exc_ 770 nm and λ_detection_ = 525±25 nm. For the GOI setup we used 64 beam scanning at 1200 Hz, peak laser power per beam 1.56·10^5^ mW, time-gate 400 ps, gain 800V, 20 to 40 data points on the fluorescence decay, exposure time = 218 ms, image size 200x200 µm² (571x571 pixel). For the p-TCSPC setup we employed single beam scanning at 1200 Hz, peak laser power per beam 1.6·10^5^ mW, time window 80 ps, 157 data points, gain 230 V, image size 200x200 µm² (512x512 pixel), multiple scanning = 3. 3D images were acquired with a step-size along the optical axis (z axis) of 1 µm.

3D time-resolved fluorescence images in hippocampus slices of *Thy1 EGFP* mice were acquired both with the GOI and the p-TCSPC setup at λ_exc_ 920 nm and λ_detection_ = 525±25 nm. For the GOI setup we used 64 beam scanning at 800 Hz, peak laser power per beam 3.6·10^5^ mW, time-gate 400 ps, gain 800V, 21 data points on the fluorescence decay, exposure time = 322 ms, image size 300x300 µm² (430x430 pixel). For the p-TCSPC setup we employed single beam scanning at 1200 Hz, peak laser power per beam 3.13·10^5^ mW, time window 160 ps, 78 data points, gain 230 V, image size 300x300 µm² (512x512 pixel), multiple scanning = 3. The z step-size for the acquisition of 3D images was 2 µm.

**FLIM experiments in hippocampus slices**

3D time-resolved fluorescence images in hippocampus slices of *CerTN L15* mice were acquired both with the GOI and the p-TCSPC setup at λ_exc_ 850 nm and λ_detection_ = 475±20 nm. For the GOI setup we used 32 beam scanning at 800 Hz, peak photon flux *_Ø_* = 2.18·10^30^ photons/s·cm², time-gate 400 ps, gain 800V, 40 data points on the fluorescence decay, exposure time = 236 ms, image size 200x200 µm² (492x492 pixel). For the p-TCSPC setup we employed single beam scanning at 800 Hz, peak photon flux *_Ø_* = 2.18·10^30^ photons/s·cm², time window 80 ps, 157 data points, gain 230 V, image size 200x200 µm² (512x512 pixel), multiple scanning = 15. 3D images were acquired with a z step-size of 4 µm. The acquisition time per time-resolved image was 20 s on the GOI detector and 24 s on the p-TCSPC detector.

**Intravital FLIM experiments**

3D time-resolved fluorescence images in the brain stem of anesthetized *CerTN L15* mice affected by EAE were acquired with the p-TCSPC setup at λ_exc_ 850 nm and λ_detection_ = 475±20 nm. For the p-TCSPC setup we employed single beam scanning at 800 Hz, peak photon flux *_Ø_* = 3.28·10^30^ photons/s·cm² (peak laser power 7.83·10^5^ mW), time window 160 ps, 78 data points, gain 230 V, image size 150x150 µm² (256x256 pixel), multiple scanning = 3. 3D images were acquired with a z step-size of 2 µm between 80 and 150 µm depth. The acquisition time per time-resolved image was 7.5 s or 12 s on the p-TCSPC detector.

**Intravital FLIM experiments: photobleaching and photodamage**

In these experiments, the excitation wavelength was 850 nm and the fluorescence was detected at 475±20 nm. 3D fluorescence data sets of 150x150x12 µm³ (512x512x7 voxel) were recorded every 60 or 90 s. The applied peak laser power was 2.93·10^5^ mW. Multiple scanning of 2 or 3 times at 800 Hz frequency was employed.

**III. Supplemental data analysis**

The effect of the instrument response function (IRF) of the p-TCSPC setup (*Fig. S2*) and of the time gate of the high-resolution intensifier (GOI) setup (*Fig. S2*) have been considered for the non-linear evaluation, i.e. reconvolution with the IRF was performed. Due to the fact that both IRF and time-gate of the FLIM field-detector are symmetric, the benefit of reconvolution falls under the error margins.

Image representation and 3D image reconstruction was performed with Volocity 5.2 (Perkin Elmer, Germany) and with ImageJ. Graph representation was made with Origin (OriginLab, CA, US). All the FLIM data acquired by means of the hybrid-detector based single-channel TCSPC (SPC-150, Becker&Hickl) were evaluated with software of the same company (*Fig. S4*).

**FLIM in brain slices and in the central nervous system of *CerTN L15* mice**

A Levenberg-Marquardt algorithm was used for biexponential evaluation: I(t) = a_1_·e^-t/τ^ _1_ + a_2_·e^-t/τ^ _2_ + ε with I(t) the time-resolved fluorescence signal of Cerulean, τ_1_ and a_1_ the fluorescence lifetime and fraction of FRET-quenched Cerulean, respectively, τ_2_ and a_2_ the fluorescence lifetime and fraction of unquenched Cerulean, respectively, and ε background. To determine the fluorescence lifetimes of FRET-quenched and unquenched Cerulean, we applied a 3x3 Gaussian filtering to the intensity images. For standard evaluation we used global analysis of the time-resolved data relying on the previously determined fluorescence lifetimes of Cerulean.

**References**

1. Niesner R, Andresen V, Neumann J, Spiecker H, Gunzer M (2007) The power of single and multibeam two-photon microscopy for high-resolution and high-speed deep tissue and intravital imaging. Biophys J 93: 2519-2529.

2. Niesner RA, Andresen V, Gunzer M (2008) Intravital two-photon microscopy: focus on speed and time resolved imaging modalities. Immunol Rev 221: 7-25.

3. Wahl M, Rahn HJ, Rohlicke T, Kell G, Nettels D, et al. (2008) Scalable time-correlated photon counting system with multiple independent input channels. Rev Sci Instrum 79: 123113.

4. Soloviev VY, Tahir KB, McGinty J, Elson DS, Neil MAA, et al. (2007) Fluorescence lifetime imaging by using time-gated data acquisition. Applied Optics 46: 7384-7391.

5. Heim N, Garaschuk O, Friedrich MW, Mank M, Milos RI, et al. (2007) Improved calcium imaging in transgenic mice expressing a troponin C-based biosensor. Nat Methods 4: 127-129.

6. Feng G, Mellor RH, Bernstein M, Keller-Peck C, Nguyen QT, et al. (2000) Imaging neuronal subsets in transgenic mice expressing multiple spectral variants of GFP. Neuron 28: 41-51.

7. Luche H, Weber O, Nageswara Rao T, Blum C, Fehling HJ (2007) Faithful activation of an extra-bright red fluorescent protein in "knock-in" Cre-reporter mice ideally suited for lineage tracing studies. Eur J Immunol 37: 43-53.

8. Siffrin V, Brandt AU, Radbruch H, Herz J, Boldakowa N, et al. (2009) Differential immune cell dynamics in the CNS cause CD4+ T cell compartmentalization. Brain 132: 1247-1258.

9. Siffrin V, Radbruch H, Glumm R, Niesner R, Paterka M, et al. (2010) In vivo imaging of partially reversible th17 cell-induced neuronal dysfunction in the course of encephalomyelitis. Immunity 33: 424-436.
